# Multiple network properties overcome random connectivity to enable stereotypic sensory responses

**DOI:** 10.1038/s41467-020-14836-6

**Published:** 2020-02-24

**Authors:** Aarush Mohit Mittal, Diksha Gupta, Amrita Singh, Andrew C. Lin, Nitin Gupta

**Affiliations:** 1 Department of Biological Sciences and Bioengineering, Indian Institute of Technology Kanpur, Kanpur, Uttar Pradesh 208016 India; 2 Princeton Neuroscience Institute, Princeton University, Princeton, NJ 08544 USA; 3 Janelia Research Campus, Howard Hughes Medical Institute, Ashburn, VA 20147 USA; 4 Department of Biomedical Science, University of Sheffield, Firth Court, Western Bank, Sheffield, S10 2TN UK

**Keywords:** Neuroscience, Olfactory system, Neurophysiology

## Abstract

Connections between neuronal populations may be genetically hardwired or random. In the insect olfactory system, projection neurons of the antennal lobe connect randomly to Kenyon cells of the mushroom body. Consequently, while the odor responses of the projection neurons are stereotyped across individuals, the responses of the Kenyon cells are variable. Surprisingly, downstream of Kenyon cells, mushroom body output neurons show stereotypy in their responses. We found that the stereotypy is enabled by the convergence of inputs from many Kenyon cells onto an output neuron, and does not require learning. The stereotypy emerges in the total response of the Kenyon cell population using multiple odor-specific features of the projection neuron responses, benefits from the nonlinearity in the transfer function, depends on the convergence:randomness ratio, and is constrained by sparseness. Together, our results reveal the fundamental mechanisms and constraints with which convergence enables stereotypy in sensory responses despite random connectivity.

## Abstract (web summary)

Because of stochastic connections between some brain regions, an identified neuron can receive different inputs across individual animals and yet respond similarly to sensory stimuli. Here the authors reveal the network mechanisms that enable stereotypic sensory responses across individuals.

## Introduction

Is your red the same as my red? This question and its variations have puzzled humans for generations. Modern neuroscience offers a way to answer a more tractable form of this question: does your brain generate the same neural activity in response to a red stimulus as my brain does? Experiments have shown that neurons in some brain areas have hard-wired connections, identical across individuals of the same species, and consequently also have stereotypic neural responses across individuals^1–5^. However, neurons in some areas show variability in their synaptic partners across individuals^6–8^. Can brain areas receiving inputs through non-stereotypic connections generate stereotypic responses?

The insect olfactory system provides an ideal system to examine this question because of its well-characterized circuitry in the first four layers of sensory processing, and individually identifiable neurons. Olfactory sensory neurons located on the antennae or palps connect to projection neurons (PNs) in the antennal lobe. These connections are highly stereotyped across individuals^9–13^. Expectedly, the responses of PNs to odors are also stereotyped^14,15^. Axons of PNs form synapses with Kenyon cells (KCs) in the mushroom body. Anatomical studies suggest that, unlike the connections at the previous layer, the PN-KC connections are random and non-stereotyped across individuals^8,16–18^, and intracellular recordings found no stereotypy in the responses of KCs^16^. KCs send their output to a small population of mushroom body output neurons (MBONs); in *Drosophila melanogaster*, there are 34 MBONs in all, belonging to 21 morphological types^19^. Hige et al.^20^ measured the odor responses of identified MBONs in different flies and found that MBONs responses across individuals were not identical but many of the MBONs had significantly more correlations across individuals than expected by chance. How do these MBONs generate stereotypic responses, when their input comes from KCs with non-stereotypic responses? Although mushroom bodies have been traditionally viewed as responsible for learning and memory, recent studies show that some MBONs are also involved in innate behaviors^21–23^, and thus consistent responses across animals for untrained stimuli are desirable.

Schaffer et al.^24^ looked at a similar question in the vertebrate olfactory system, where mitral cells and piriform cortex neurons have connectivity and responses analogous to PNs and KCs, respectively^25–28^. The computational model in the study exhibited stereotypy in the output of the piriform cortex when different cortices were pre-trained with the same odor, but not in the absence of such training; thus, this study implied that learning is necessary for stereotypy^24^. On the contrary, experimental data from flies showed that stereotypy increases when learning is impaired: *rutabaga* mutant flies, which are deficient in learning, show more stereotypy in MBON responses compared to wild-type flies^20^.

Here, we confirm the presence of stereotypy in MBON responses with intracellular recordings from another species, the locust *Schistocerca americana*. With network simulations constrained by the experimental data from the *Drosophila* olfactory system, we identify the factors that contribute to stereotypy. We show that stereotypy is a natural consequence of convergence following random connectivity and does not require learning. These observations from simulations are confirmed by deriving a closed-form expression for stereotypy in an analytical model. The simulations also predict an antagonism between sparseness and stereotypy, which we test using in vivo recordings from locusts and flies. Our results reveal the fundamental mechanisms and constraints that determine the level of stereotypy in any neural network with random connections.

## Results

### Experimental evidence for stereotypy using two metrics

**Fig. 1:**
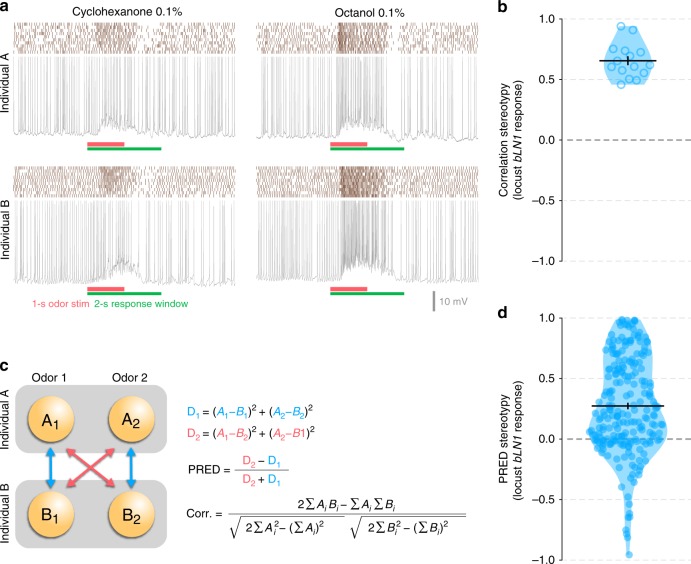
Stereotypy in locust MBON responses confirmed with two metrics. **a** Raster plots with representative recordings showing responses of the locust *bLN1* MBON in two individuals to two odors. Odor was presented for 1 second (red bar) and the spikes in the responses were counted in a 2-s window following odor onset (green bar); scale bar for recordings, 10 mV. In this sample, the responses appear to be stereotypic, with more spikes for octanol 0.1% and fewer spikes for cyclohexanone 0.1*%* in both the individuals. **b** Violin plot showing the value of correlation stereotypy in *bLN1* responses in the locust dataset. Each point represents the Pearson’s correlation coefficient, calculated using the 6-length vector (responses to 6 odors) for a pair of individuals (*n* = 15 pairs of individuals). **c** Schematic description of the PRED metric for stereotypy and comparison with the correlation metric. **d** PRED stereotypy in *bLN1* responses in the locust dataset used in (**b**). Note the bias towards positive values. Each point represents the pairwise PRED stereotypy value, calculated for a pair of odor responses in a pair of individuals, giving a total of 225 values (15 pairs of odors × 15 pairs of individuals). In **b**, **d** black horizontal line represents the mean. Error bars represent s.e.m.

As stereotypy has been examined in only *Drosophila* MBONs so far^20^, we first checked whether MBONs in other species also have stereotypic responses. Like flies, locusts have multiple classes of MBONs, of which the class *bLN1* has only one neuron per hemisphere and therefore can be uniquely identified across individuals; in terms of firing rates or response probabilities, *bLN1* is not very different from other classes of MBONs, all of which respond broadly (~97% probability) to odors^29^. We analyzed a dataset of *bLN1* responses (see Methods; Fig. 1a and Supplementary Fig. 1a and b) to 6 odors in 6 individuals^29^. In previous studies, response stereotypy of a neuron has been quantified as the Pearson’s correlation coefficient between its response vectors in two individuals, where each vector contains the trial-averaged responses of an individual to a given set of odors^20,24^. We found that the average correlation between the *bLN1* responses across individuals was 0.66 (Fig. 1b), significantly greater than the chance level of 0 (*P* = 4.58 × 10^−11^, *n* = 15 pairs of individuals, *t*-test). Thus, *bLN1* responses in locusts are stereotyped across individuals; together with the previous observations in *Drosophila*^20^, this finding suggests that response stereotypy in MBONs, despite their random connectivity, is likely to be a general property across species.

The stereotypy in the responses of a neuron indicates how similar or consistent the sensory responses of that neuron are in different individuals; this response stereotypy would make behavioral responses across individuals more consistent for the same inputs. It is important to note that, in this context, the stereotypy is a characteristic of a type of response (e.g., the olfactory response of a particular neuron or the total olfactory response in a brain region), and not a property of specific odors or specific individuals, even though in practice it is estimated by looking at the responses to a limited set of odors in a limited set of individuals. Correlation is just one of the ways in which the response similarity across individuals can be quantified. Using correlation as a metric for stereotypy captures whether the relative responses to different odors follow similar patterns in individuals but ignores the absolute differences in the responses between the individuals. On the other hand, using the Euclidean distance (between the response vectors of individuals) as a measure of stereotypy would capture the absolute differences but miss the information about the relative response patterns for different odors. We developed a new and simple metric for stereotypy, called pairwise relative distance (PRED), which takes into account both the absolute distances between the responses of individuals (as Euclidean distance does) as well as their relative patterns across odors (as correlation does). This metric is calculated using data of pairs of individuals for pairs of odors, and essentially quantifies whether individual A’s response to odor 1 is closer to individual B’s response to odor 1 than to B’s response to odor 2 (Fig. 1c; see Methods). In other words, PRED stereotypy quantifies whether the responses of two individuals differ more for different odors than for the same odor.

PRED relates to the idea of odor discriminability or classification accuracy, but differs in an important way: conventional metrics for odor discrimination or classification measure how separable the responses to different odors are within an individual; on the other hand, PRED stereotypy measures how separable the responses to different odors are across individuals. If the response is stereotyped, it should be possible to predict whether a given response value in individual A belongs to odor 1 or odor 2, by comparing it to the known responses of individual B to the same two odors—this is feasible when the response of individual A to odor 1 is more similar to individual B’s response to odor 1 than to B’s responses to odor 2, and A’s response to odor 2 is more similar to B’s response to odor 2 than to B’s response to odor 1. PRED stereotypy, like correlation stereotypy, ranges between −1 and 1, where 1 indicates a perfectly stereotyped response (i.e., identical responses across individuals), 0 indicates no stereotypy (e.g., unrelated responses), and −1 indicates the case when two individuals have different responses to the same odor but similar responses to different odors. If response data are available for more than two odors or more than two individuals, the metric can be calculated for all possible pairs and then averaged to get the final value.

We calculated PRED stereotypy in the locust *bLN1* responses using all combinations of the 15 pairs of odors and 15 pairs of individuals, and found it also to be significantly above the chance level of 0 (mean = 0.27, *P* = 2.25 × 10^−20^, *n* = 225 combinations, *t*-test; Fig. 1d; random resampling test, *P* < 0.001). Thus, both metrics confirm the presence of stereotypy in locust MBON responses. These observed stereotypy values were smaller than those seen in PNs (Supplementary Fig. 1c; see Methods), but much higher than those seen in genetically labeled KCs (Supplementary Fig. 1d; see Methods) in *Drosophila*.

### Stereotypy originates in the KC population

**Fig. 2:**
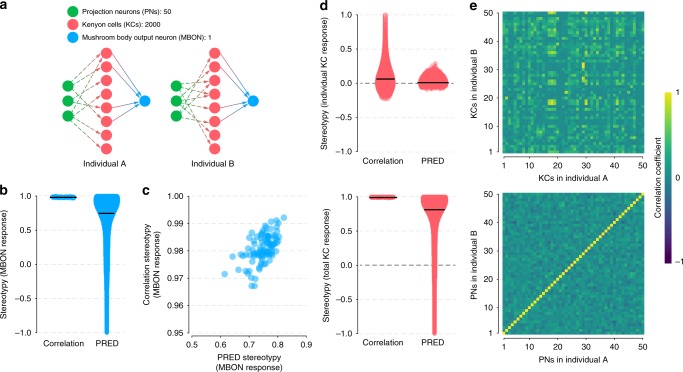
Stereotypy in model MBONs and KCs. **a** Schematic representation of the simulated mushroom body networks in two different individuals. Projection neuron (PN, green) to Kenyon cell (KC, pink) connections are random and vary across individuals. The number of KCs connected to the mushroom body output neuron (MBON, blue) was kept same across individuals. **b** Correlation stereotypy and PRED stereotypy in MBON response in a realistic network with random PN-KC connections across individuals; for correlation, *n* = 100 points corresponding to different network iterations with different random seeds; for PRED, *n* = 495000 points (100 iterations × 950 odor pairs from 100 odors). **c** Scatter plot of correlation stereotypy versus PRED stereotypy in MBON response for the same simulations as in (**b**) shows that both metrics behave similarly. The PRED stereotypy is averaged over all 4950 odor pairs within a network iteration (*n* = 100 iterations). **d** (top) Correlation stereotypy and PRED stereotypy in individual KC response for the same simulations as in (**b**). Both metrics confirm the absence of stereotypy in individual KCs, *n* = 100537 KCs that responded to at least one odor in both individuals, out of the 200,000 KCs from 100 network iterations. (bottom) Correlation stereotypy and PRED stereotypy in total KC response for the same simulations as in (**b**); number of data points same as in (**b**). **e** Correlation between the responses of the PNs (bottom) and the KCs (top) in two individuals in a simulation. The correlation is calculated using the 100-length vector (responses to 100 odors) for each pair of PNs (50 in total) or for each pair of KCs among the first 50 KCs (by spatial ordering) that responded to at least one odor in both individuals.

The observations made above in locusts, along with previous observations in *Drosophila*^20^, suggest that MBON stereotypy may be a general phenomenon among insects, as the basic organization of the olfactory circuit is similar across species. To understand the origin of stereotypy in insect MBONs, we constructed a computational model of the inputs received by an MBON (see Methods and Fig. 2a), constrained with parameters taken from the more widely studied *Drosophila* system. The model included three layers of neurons: an input layer with 50 PNs, each corresponding to an olfactory glomerulus^8,30,31^; a middle layer of 2000 KCs^32^; and an output layer with the MBON. Although there are ~160 PNs in *Drosophila*^33,34^, PNs that innervate the same glomerulus have similar morphology^13,35,36^ and similar responses to odors^15,16,37^. Previous recordings have shown that each odor activates a unique set of PNs, such that any PN responds to a given odor with about 50% probability and an odor-specific spiking rate^15,38^. We set the threshold of KCs such that only ~10% of KCs responded to any given odor, to mimic the sparse responses observed in these neurons experimentally^38,39^. The MBON was connected to half of the KCs^40^. With these settings, the model MBON responded to all odors, in agreement with experimental reports^20,29^. We simulated two networks, corresponding to two individuals, and measured the response of each network to a hundred different odors.

As a positive control and a simple verification of our analysis procedures, we first confirmed that MBON responses were stereotyped in a modified (and unreal) network with identical connections between PNs and KCs across individuals, even in the presence of noise (Supplementary Fig. 2a). As a negative control, we verified that stereotypy was not seen in the MBON response if the PN responses in the two individuals were made non-stereotypic (Supplementary Fig. 2a). We then analyzed the real network with non-stereotypic PN-KC connections and stereotypic PN responses across individuals (Fig. 2a). Even with the random connections, the MBON showed a surprisingly high stereotypy: correlation stereotypy was 0.98 (*P* = 7.45 × 10^−228^, *n* = 100 network iterations, *t*-test; Fig. 2b) and PRED stereotypy was 0.75 (*P* = 1.22 × 10^−131^, *n* = 100; Fig. 2b), with both metrics behaving similarly (Pearson correlation, *r* = 0.57, *P* = 5.06 × 10^−10^; Fig. 2c). These values are higher than experimentally measured values of stereotypy probably because biological and experimental noise reduces stereotypy; adding noise to the inputs of neurons in our simulations indeed reduced the stereotypy (Supplementary Fig. 2b; the distribution of stereotypy values in the rightmost panel is comparable to the distribution of experimentally measured PRED stereotypy shown in Fig. 1d). Robust stereotypy values were obtained even as we varied the number of PNs or their response probability in our simulations, indicating that the observation of stereotypy is not limited to a narrow range of input parameters (Supplementary Fig. 2c and d; other network parameters are analyzed in later sections).

It was not immediately clear what led to stereotypic responses in the MBON in our simulations, since the individual KCs driving its activity showed extremely low stereotypy (correlation stereotypy: 0.0616 ± 0.1478 (s.d.); PRED stereotypy: 0.0084 ± 0.0201; *n* = 100537 KCs that responded to at least one odor in both individuals, out of the 200,000 KCs from 100 network iterations; Fig. 2d top). Spatially identical KCs (having the same index in the list of KCs) in two different individuals were no more correlated to each other than to other KCs (Fig. 2e top), in agreement with experimental observations^16,20,41^; contrast this with the high correlation between identical PNs in different individuals (Fig. 2e bottom). Since the MBON receives converging inputs from multiple KCs, we looked at stereotypy in the total KC response (the sum of the KC spiking rates). The total KC response revealed high stereotypy with both metrics (correlation stereotypy = 0.99, *P* = 2.36 × 10^−254^; PRED stereotypy = 0.81, *P* = 1.46 × 10^−152^; Fig. 2d bottom), in agreement with experimental data^20^. These results confirm that stereotypy is already present in the total response of the KC population, even if not observed in the responses of individual KCs. Given that an MBON receives input from a large fraction of the KC population^19^, the presence of stereotypy in the KC population can explain the stereotypy in the MBON. Henceforth, we focus on understanding the origin of stereotypy in the KC population.

### Stereotypy does not require learning

**Fig. 3:**
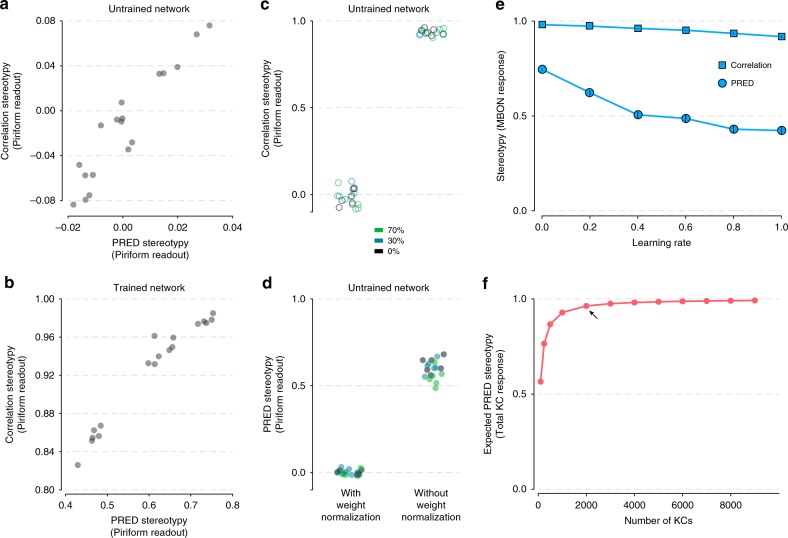
Stereotypy does not require learning. **a**, **b** Comparison of PRED stereotypy with correlation stereotypy in the responses of the readout neuron in the simulations of the untrained (**a**) or trained (**b**) piriform network using code provided by Schaffer et al.^24^. **c**, **d** Stereotypy in the simulations of the untrained piriform network, with and without the weight normalization step calculated using both correlation (**c**) and PRED (**d**) metrics. The three different colors represent simulations with different levels of overlap among the olfactory bulb responses to odors as described by Schaffer et al.: 70% overlap (green), 30% overlap (blue) and 0% overlap (gray). **a**–**d** have 18 data points each (6 iterations × 3 sets of odors). **e** Stereotypy in MBON response reduces with the learning rate, as seen in simulations with 100 odors and 2 individuals. Learning of an odor resulted in either increasing or decreasing the number of synapses between the MBON and the KCs activated by that odor. Half of the odors, selected randomly for each individual, were learnt sequentially, and each learning event resulted in increase or decrease in synapses with equal probability. When the synapses were increased, of all the KCs that were activated by the learned odor and were not connected to the MBON, a certain fraction (indicated by the learning rate) were connected to the MBON. When the synapses were reduced, of all the KCs that were activated by the learned odor and were connected to the MBON, the same fraction were disconnected from the MBON. The MBON responses for both individuals were recalculated after modifying the synapses, *n* = 100 iterations. Error bars represent s.e.m. **f** Analytical model proved the existence of stereotypy in absence of learning. Plot of stereotypy versus the number of KCs suggested a dependence of stereotypy on the size of the network, as later seen in the simulations (compare to Fig. 6d). Each value is calculated using the formulae derived in the analytical model (see Supplementary equations). Arrow indicates the default value (matching the *Drosophila* system).

Piriform cortices in vertebrates are analogous to insect mushroom bodies and connect randomly with their input neurons^26^. A recent modeling study by Schaffer et al.^24^ concluded that learning is necessary for stereotypy across different piriform cortices. In their study, the responses of the output neurons of two different piriform cortices were uncorrelated unless the two models were trained with a common odor to set the synaptic weights between piriform cortical neurons and their outputs. Our results, however, show that stereotypy exists even in a simple network lacking any form of learning. This difference is unrelated to the metrics used for stereotypy; the correlation metric, used by Schaffer et al.^24^, and the PRED metric gave similar results for both trained and untrained networks in their simulations (Fig. 3a, b). Rather, we found that the difference arose mainly because Schaffer et al.^24^ used a normalization of weights in their simulations: the weights of all the incoming synapses to an MBON were scaled to make the mean weight zero, converting many of the positive synaptic weights into negative weights. Removing this normalization (see Methods) produced high stereotypy (in both correlation and PRED metrics) even without learning in their model (Fig. 3c, d), confirming our conclusion that learning is not necessary for stereotypy. (Removing the normalization did not reduce the signal-to-noise ratio; Supplementary Fig. 3). Our results are also more consistent with experiments showing high stereotypy in learning-impaired *rutabaga* mutant flies^20^. Although MBONs receive stereotyped inputs from KCs^19^, learning can modify the connections between KCs and MBONs^42–46^, making the inputs to an MBON different across individuals. Indeed, when we incorporated learning in our model by increasing or decreasing the numbers of synapses between the MBON and the set of KCs responding to a learned odor in an individual, the MBON stereotypy decreased (Fig. 3e), in agreement with the experimental data^20^. Taken together, these results strongly suggest that learning is not necessary for stereotypy; rather, learning is antagonistic to stereotypy.

### PRED versus correlation as metrics for stereotypy

In the analyses of locust *bLN1* experimental data, our simulations, and the simulations of Schaffer et al.^24^, both the correlation and the PRED metrics gave similar results. However, we note that there are certain areas in which PRED offers advantages over correlation. First, if the dataset includes only two odors, the correlation metric gives only extreme values (−1 or 1) regardless of the response magnitudes, whereas the PRED metric provides a graded quantification for high-stereotypy and low-stereotypy scenarios, as shown in Supplementary Fig. 4a. If an individual responds the same to both odors (as in the green shaded example in Supplementary Fig. 4a), the correlation is undefined while PRED provides an appropriate value of 0. Second, as stereotypy is considered a property of a neuron and not of the odors or the individuals whose data is used to estimate it, the value of stereotypy should not be systematically biased by the number of odors or the individuals available. When we varied the number of individuals in our simulations, both metrics gave unvarying estimates for stereotypy (Supplementary Fig. 4b); this result is expected as both metrics are calculated on pairs of individuals and then averaged. However, when we varied the number of odors, the PRED stereotypy did not vary but the correlation stereotypy systematically increased in magnitude, suggesting that the correlation metric is biased by the number of odors available in a dataset (Supplementary Fig. 4c). Similarly, the correlation stereotypy values obtained from two non-overlapping sets of odors were systematically smaller than the stereotypy obtained from the combined dataset; PRED stereotypy did not change (Supplementary Fig. 4d). These observations suggest that PRED is a more robust measure for stereotypy than correlation, especially when the number of odors is small. In simulations with 100 odors, PRED values were not affected much by exclusion of random odors, and reduced only slightly if odors with the lowest or the highest activity were excluded (Supplementary Fig. 4e). Another advantage of the PRED stereotypy metric, as we show next, is that its relatively simple form makes it amenable to analytical modeling. Note that simulations with two odors and two individuals also showed robust PRED stereotypy (Supplementary Fig. 4b). Henceforth, we use PRED as the default metric for stereotypy, and use two odors and two individuals in the simulations.

### Theoretical model of stereotypy

There is no analytical model available yet for understanding stereotypy without running the simulations. To complement the findings from our simulations, we developed an analytical model to calculate the expected value of PRED stereotypy in a simplified network with no learning mechanism. We considered a network of binary PNs and KCs connected to each other by random connection matrices (see Supplementary equations). We then derived a formula for the expected value of stereotypy in the total KC response by considering two random odors and two random individuals at a time. The formula confirms the presence of stereotypy even in this simple network without any learning (0.96; Supplementary Fig. 5a), providing theoretical support to the findings from the experiments and the simulations. Further, the calculations show that the stereotypy increases with the number of KCs (Fig. 3f), providing a clue for network parameters that are important for stereotypy. The calculations also show that the stereotypy remains high even if the PN-KC connection probability or the number of PNs in the model are varied over a wide range (Supplementary Figs. 5b and c). Below we use simulations to further investigate the contributions of various properties of the network to stereotypy.

### Multiple features of PN responses contribute to stereotypy

**Fig. 4:**
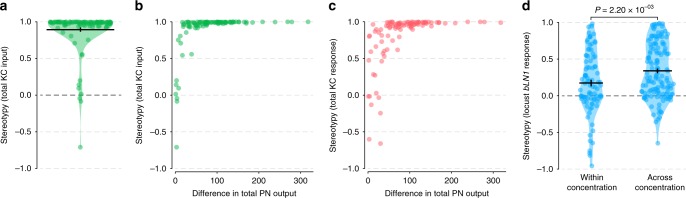
Difference in input drives affects stereotypy. **a** Stereotypy in total KC input in the simulations. PRED metric was used for calculation of stereotypy in these and all subsequent analyses. **b**, **c** Scatter plots of stereotypy in total KC input (**b**) or total KC response (**c**) versus the difference in total PN outputs between the two odors. **a**–**c** Each point represents a network iteration (*n* = 100). **d** Stereotypy in locust *bLN1* responses is more for the across-concentration group than the within-concentration group. The within-concentration group has *n* = 90 stereotypy values (15 pairs of individuals × 3 pairs of odors × 2 concentration levels) and the across-concentration group has *n* = 135 stereotypy values (15 pairs of individuals × 9 pairs of odors such that one odor in the pair is at 0.1% concentration and the other odor is at 10% concentration). In **a**, **d** error bars represent s.e.m.

We first studied how the inputs received by the KCs affect their stereotypy. Stereotypy in the KC population did not depend on the correlation between the PN response profiles of different odors, as any such correlations are discarded by the random connectivity at the next level (Supplementary Fig. 6a). Hige et al.^20^ speculated that the total input drive to the KCs (i.e., the total output of PNs) could be a characteristic of each odor. In flies, it has been observed that increasing odor concentration increases PN activity, although not steeply because of lateral processing within the antennal lobe^47,48^. In locusts, higher odor concentrations have been shown to generate more synchrony among PN responses^49^, which could also make the PN drive more effective in activating KCs. How would the differences in input drives affect the stereotypy? Differences in the total PN output generated by two odors would translate into differences in (a) the total inputs received by the KCs and (b) the total response produced by the KCs. We first looked at the total KC input, calculated as the sum of inputs received by each KC (so that PNs that are connected to more KCs contribute more to the total), and found that it was stereotyped (0.89, *P* = 1.42 × 10^−53^; Fig. 4a). Further, as the difference in the total input drives (the total number of spikes in the PN population) increased, stereotypy also increased in the total KC input (Pearson correlation, *r* = 0.43, *P* = 6.64 × 10^−06^; Fig. 4b) as well as in the total KC response (*r* = 0.50, *P* = 9.33 × 10^−08^; Fig. 4c).

These simulations predict that stereotypy for a pair of odors should be higher when the drives generated by the two odors differ more. To test this prediction, we used the locust *bLN1* dataset, which included responses for two different concentrations (0.1 and 10%) of three odorants. We divided the pairwise stereotypy values from the locust dataset into two groups: a within-concentration group, including values calculated for pairs of odors at the same concentration; and an across-concentration group, including values calculated for pairs of odors at different concentrations (that is, with one odor in the pair at 0.1% and the other at 10%). The across-concentration group is expected to have more differences in the input drives between the two odors in a pair, and therefore more stereotypy. The experimental data confirmed this prediction: the within-concentration group had a stereotypy of 0.17 which was significantly less than 0.34 seen in the across-concentration group (*P* = 2.20 × 10^−03^, unpaired *t*-test; Fig. 4d).

**Fig. 5:**
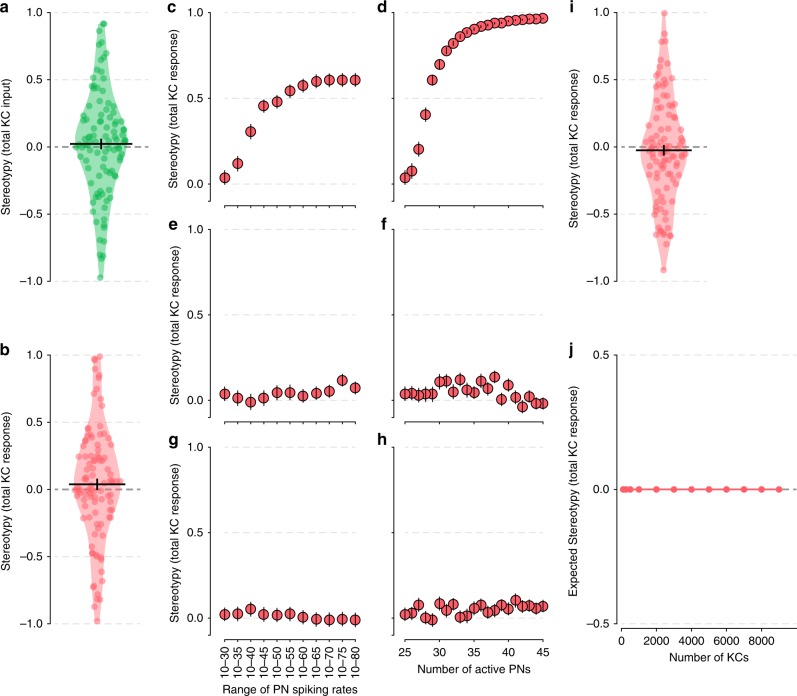
Stereotypy in simulations with the same input drives for both odors. **a**, **b** Stereotypy in total KC input (**a**) and total KC response (**b**) when the total input drives to both odors are equal. **c** Stereotypy in total KC response versus the range of PN spiking rates when the range of spiking rates is changed to the indicated values for one odor while it is maintained at 10–30 for the other. **d** Stereotypy in total KC response versus the number of active PNs when the number is changed to the indicated values for one odor while it is maintained at 25 for the other. **e** Similar to (**c**) except that the range of PN spiking rates is changed for both odors. **f** Similar to (**d**) except that the number of active PNs is changed for both odors. **g**, **h** Plots similar to (**c**) and (**d**), respectively, but with the modification that a linear transfer function, *y*_*i*_ = *mx*_*i*_ − *t*, is used to generate KC responses from their inputs. Here, *y*_*i*_ is the response of the *i*^*th*^ KC, *x*_*i*_ is the input to the *i*^*th*^ KC, *t* is the threshold, and *m* is chosen such that the total KC responses are equal to those seen in the default simulations. **i** Stereotypy in total KC response when the PN responses to the second odor are generated by shuffling the PN labels in the response to the first odor. In this condition, the total PN output, the number of active PNs, and the spiking range of active PNs are identical for the two odors. **j** Analytical model confirmed that there is no stereotypy, regardless of the number of KCs, when the PN responses to the two odors are shuffled versions of each other. In all panels, *n* = 100 iterations; error bars represent s.e.m.

Although stereotypy was positively correlated with differences in input drives in our simulations, these correlations were small (0.43 and 0.50 for stereotypy in total KC input and total KC response, respectively), and there was high stereotypy in some simulations with little difference in input drives (Fig. 4b, c). These results indicated that the odor-specificity of the total PN drive is not the sole contributor to stereotypy. The number of active PNs and the range of spiking rates of these PNs show some variation across odors^15,38^. We reasoned that these two factors could also contribute to stereotypy. To measure the effect of these two factors independently, we first constrained the total input drives to be the same for both odors (see Methods). In this case, there was no stereotypy in the total KC input (0.02, *P* = 0.5763; Fig. 5a) or the total KC response (0.04, *P* = 0.3692; Fig. 5b). Then we made either the number of active PNs or their spiking rate range different for the two odors. Stereotypy increased on increasing the spiking rate range of PNs for one odor while keeping it at the default value for the other odor (Fig. 5c). Similarly, the stereotypy increased when we increased the number of active PNs for one odor while keeping it at the default value for the other odor (Fig. 5d). This increase in stereotypy was not simply due to larger values of the parameters but due to the differences in their values between the two odors: stereotypy did not increase when we increased the parameters for both odors in tandem (Fig. 5e, f). Thus, even with the same total input drives, the KC population could get stereotyped using the differences in the spiking rate ranges or the number of active PNs across odors. These two factors may be important given that lateral processing within the antennal lobe tends to equalize the total PN drives to different odors^48,49^.

In these simulations with fixed input drives, while stereotypy was seen in the total KC response, it was absent in the total KC input (Supplementary Fig. 6b and c). This observation suggested that the non-linearity introduced by the response threshold in the generation of KC responses from their inputs may be required for stereotypy in the absence of differences in input drives across odors. To confirm this, we ran the simulations with fixed input drives but differences in the number of active PNs or spiking rate ranges with a linear transfer function for KCs; in these simulations, the stereotypy was lost (Fig. 5g, h), showing the role of the non-linearity in maintaining stereotypy in the absence of differences in input drives.

It is intuitively clear that there should be no stereotypy if different odors generate the same PN responses. What is the minimal set of parameters that must differ between PN responses to generate stereotypy? Our results show that when odors elicit the same numbers of spikes across the PN population, activate the same numbers of PNs, and result in the same range of PN spiking rates, there is no stereotypy in the total KC response. This provides a lower bound: the PN population responses across odors must differ in at least one of these three parameters for the existence of stereotypy (Fig. 5c, d**;** Supplementary Fig. 7). These results also predict that if PN responses elicited by a given odor can be obtained by shuffling the PN labels in another odor’s responses, thereby leaving all the three parameters identical, there should be no stereotypy. Further simulations confirmed this prediction (Fig. 5i). The analytical model also confirmed that the null stereotypy is theoretically expected for shuffled PN responses and is not dependent on the specific network parameters used in our simulations (Fig. 5j; see Supplementary equations).

### Sparseness constrains stereotypy

**Fig. 6:**
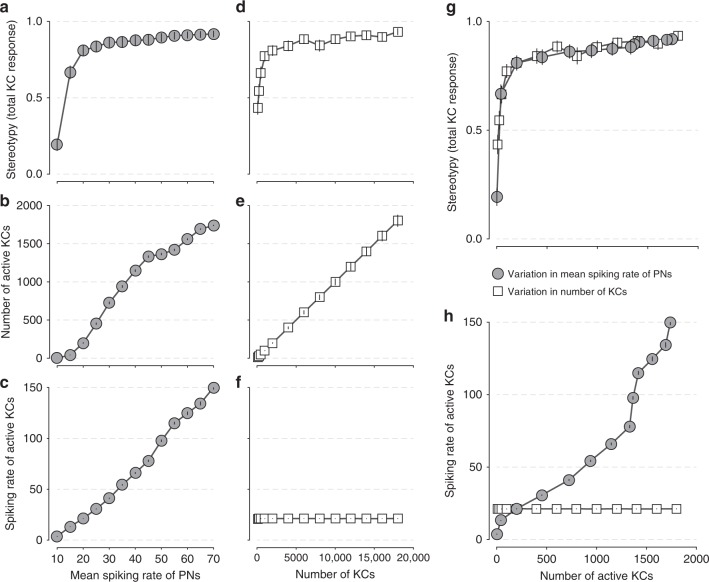
The number of active KCs but not their spiking rate determines stereotypy. **a**–**c** Stereotypy in total KC response (**a**), average number of active KCs (**b**), and the average rate of active KCs (**c**) versus the mean spiking rate of PNs. In this set of simulations, the mean spiking rate of PNs was changed by changing the PN spiking range to the indicated mean ± 10. Note that in these simulations, both the average number of active KCs and their average spiking rate increase with the increase in mean PN spiking rate. **d**–**f** Stereotypy in total KC response (**d**), average number of active KCs (**e**) and the average rate of active KCs (**f**) versus the number of KCs in the model, when this number was changed to the indicated value in a separate set of simulations. Note that in these simulations, only the average number of active KCs but not their average spiking rate increases with the number of KCs. **g**, **h** Stereotypy in total KC response (**g**) and the rate of active KCs (**h**) versus the average number of active KCs in two sets of simulations described in the previous panels. Stereotypy increased equally in both sets of simulations with the number of active KCs and did not depend on the average spiking rate of KCs, which increased in the first set but not in the second set. In all panels, *n* = 100 iterations; in each iteration, averages were taken over both odors and both individuals. Error bars represent s.e.m.

A salient feature of KCs is that very few of them respond to any given odor^39,49,50^. We asked how this sparseness in KC responses affects stereotypy. To manipulate the sparseness of KCs, we varied the mean spiking rate of PNs in our default simulations (without fixing the three factors discussed in the last section), while keeping the KC response threshold constant. Note that in the last section we analyzed the differences in PN responses between odors; here we varied the spiking rate of PNs uniformly for both odors. We found that increasing the mean spiking rate of PNs increased the stereotypy in the total KC response (Fig. 6a). This manipulation increased the net input to the KCs relative to their response threshold, an effect that can also be obtained by increasing the connection probability between PNs and KCs, or by reducing the threshold of KCs. Indeed, stereotypy increased when we increased the connection probability between PNs and KCs (Supplementary Fig. 8a) or decreased the threshold of KCs (Supplementary Fig. 8b). How does a general increase in the inputs to KCs lead to an increase in stereotypy?

Increasing the mean spiking rate of PNs increases both the average number of active KCs (Fig. 6b) and the average spiking rate of KCs (Fig. 6c). To tease apart the contributions from these two factors, we ran another set of simulations in which we increased the number of KCs in the model—this increased the average number of active KCs (Fig. 6e) without changing their average spiking rate (Fig. 6f). The average stereotypy in this case, in agreement with our analytical calculations (Fig. 3f), increased with the increasing number of KCs (Fig. 6d). In both of these simulations, the stereotypy increased equally with the increase in the number of active KCs (Fig. 6g), even though in one case the spiking rate of active KCs remained constant while in the other case it increased (Fig. 6h). Therefore, the level of stereotypy is determined primarily by the average number of active KCs and not their average spiking rate.

Our results reveal a trade-off between stereotypy and sparseness, i.e., the fraction of KCs not responding: less sparseness (more active KCs per odor) leads to more stereotypy. Although stereotypy depends on the absolute number of active KCs rather than the fraction per se, the two are equivalent in the context of a particular species’ total number of KCs. It is noteworthy that the stereotypy increases with the average number of active KCs quite sharply till this number reaches 200–300 (10–15% of 2000) and begins to saturate for larger numbers (Fig. 6g); this elbow-point near 10% response probability matches the experimentally observed levels of sparseness in KCs^38,39,50^.

### Experimental tests of the effect of sparseness on stereotypy

**Fig. 7:**
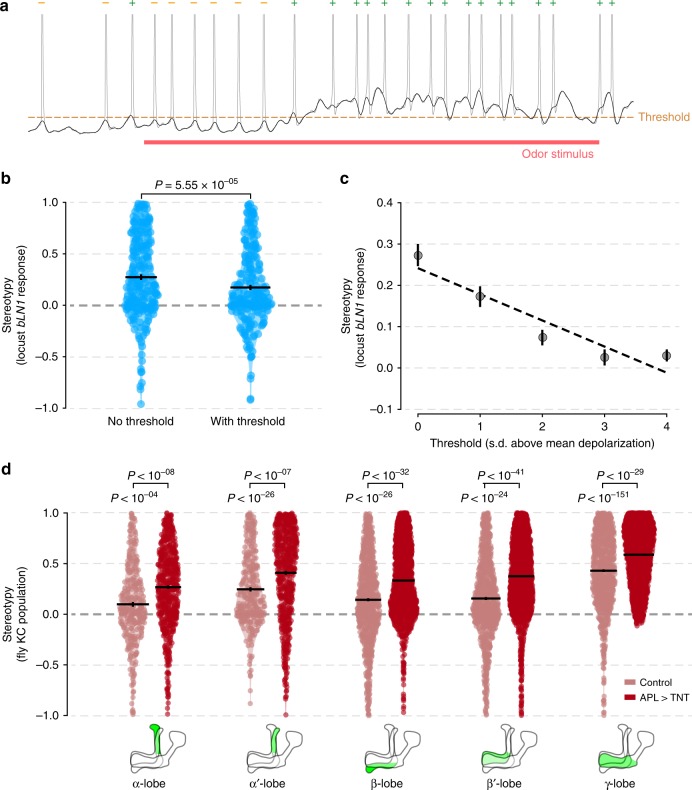
Experimental data confirm the antagonism between sparseness and stereotypy. **a** A representative intracellular recording (gray) from locust *bLN1* is shown to illustrate the method of removing spikes that occur at depolarizations below a threshold (dashed line). Discarded spikes are labeled ‘−’ and retained spikes are labeled ‘+’. The depolarization (dark black trace) is extracted by clipping spikes and filtering the recording (50-Hz low-pass). The threshold is set at one s.d. above the mean depolarization. **b** Stereotypy in locust *bLN1* response reduces when we mimic more sparseness by removing spikes below the threshold (1 s.d. above mean depolarization). **c** Stereotypy continues to reduce as sparseness is further increased by raising the threshold in multiples of s.d. above mean depolarization. In **b**, **c**, *n* = 225 values (15 pairs of odors × 15 pairs of individuals). **d** Fly KC populations imaged at different lobes of the mushroom body in control flies reveal significant stereotypy. The stereotypy further increases in all lobes as sparseness is reduced by blocking APL output (APL>TNT); *n* = 315 (control, α- and α′-lobes, from 11 hemispheres from 10 flies), *n* = 570 (APL>TNT, α- and α′-lobes, from 20 hemispheres from 14 flies), *n* = 828 (control, β-, β′- and γ-lobes, from 18 hemispheres from 15 flies), *n* = 1890 (APL>TNT, β-, β′- and γ-lobes, from 36 hemispheres from 24 flies). Error bars represent s.e.m.

We used in vivo data to test our prediction that more sparseness leads to less stereotypy. The membrane potential depolarization of an MBON reflects the synaptic input it receives from all the connected KCs. We reasoned that if the number of active KCs were reduced, the depolarization of the MBON would also reduce; consequently, the MBON would be less likely to produce spikes, particularly at those times when fewer KCs are active (that is, when the depolarization is small). Using the intracellular recordings of locust *bLN1*, we extracted the depolarization and mimicked the effect of increasing sparseness by removing spikes that occurred when the depolarization was below a threshold (Fig. 7a). Confirming our prediction, this change led to a significant reduction in stereotypy (*P* = 5.55 × 10^−05^, paired *t*-test; Fig. 7b). (The same result was obtained if we approximated increasing sparseness in KCs by reducing the number of *bLN1* spikes by a constant amount of 20 spikes; *P* = 8.4 × 10^−11^, paired *t*-test). As we increased the sparseness by further raising the threshold, the stereotypy also reduced as expected (correlation between stereotypy and threshold, *r* = −0.94, *P* = 0.017, *n* = 5 thresholds; Fig. 7c).

To test the prediction in the other direction (that is, whether less sparseness leads to more stereotypy), we used genetic manipulations in *Drosophila*. We decreased sparseness in fly KCs by blocking synaptic output from APL, a single inhibitory interneuron in the mushroom body that maintains the sparseness in the KC population^51^. We expressed tetanus toxin (TNT) in APL using an intersectional driver (see Methods) that labels APL ~60% of the time; hemispheres where APL was unlabelled served as controls. We measured stereotypy in the total responses of large sets of KCs using two-photon calcium imaging in the lobes, for a set of three odors (Supplementary Fig. 9). We found that the control hemispheres showed moderate levels of stereotypy, while hemispheres with reduced sparseness (APL>TNT) showed significantly more stereotypy (Fig. 7d). Together, experimental data from locusts and flies confirm the trade-off between sparseness and stereotypy predicted by our simulations.

### Convergence:randomness ratio determines stereotypy

**Fig. 8:**
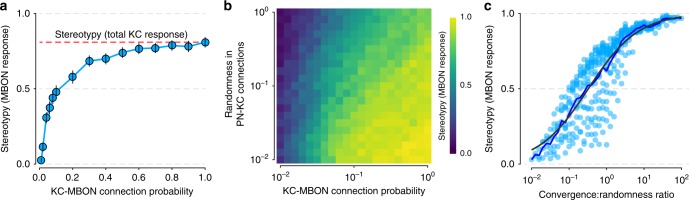
Stereotypy depends on the level of convergence. **a** Stereotypy in MBON response versus the connection probability between KCs and MBON in the model. When connection probability is 1, the MBON stereotypy matches the stereotypy in total KC response (dashed line). Error bars represent s.e.m. **b** Stereotypy in MBON response as a function of KC-MBON connection probability and the randomness in PN-KC connections (i.e., the fraction of PN-KC synapses whose values are independently set in the two individuals). Simulations were done for 21 values of each parameter in the range 0.01 to 1.0 on a log scale. **c** Stereotypy in MBON response versus the ratio of convergence (KC-MBON connection probability) to randomness (in PN-KC connections) in the data shown in (**b**). The dependence of stereotypy (*S*) on the convergence:randomness ratio (*r*) is captured well by a fit to the Hill equation, ${{S}} = \frac{{{{r}}^{0.65}}}{{0.48 + {{r}}^{0.65}}}$ (black trace); blue trace shows the average stereotypy of points with the same ratio. In all panels, *n* = 100 iterations.

If more active KCs lead to more stereotypy, MBONs receiving converging input from more KCs should generate more stereotypic responses. Indeed, we found that MBON stereotypy increased with KC-MBON connection probability (Fig. 8a) and reached the same level as KC population stereotypy when the MBON received input from all the KCs. Thus, the 21 different classes of MBONs present in *Drosophila*^19^ are expected to show different levels of stereotypy depending on the number of KCs they are connected to; this can provide one explanation for the varying levels of stereotypy found in experimental recordings of MBONs^20^. As it may be easier to measure stereotypy experimentally than to determine the synaptic connections between neurons, the high correlation between convergence and stereotypy presents a convenient experimental method for roughly estimating the number of KCs connected to an MBON, if the distributions of KC-MBON synaptic weights across different MBONs are similar.

The convergence enables the stereotypy in the MBON by overcoming the randomness in PN-KC connections. Indeed, simulations with different levels of randomness in PN-KC connections reveal that these two factors compete: in networks with lower levels of randomness, a lower level of convergence is enough to achieve stereotypy, while in networks with more randomness, a higher convergence is necessary to achieve the same level of stereotypy (Fig. 8b). In these simulations where both convergence and randomness were varied, we found that the convergence:randomness ratio as a single independent variable can provide a reliable estimate of stereotypy (*R*^2^ = 0.78; Fig. 8c; see Methods). Thus, although our simulations in previous sections assumed complete randomness in the connections between PNs and KCs, the results here show that the stereotypy will be higher, or can be maintained with lower levels of convergence, when the connections are only partially random.

## Discussion

In summary, our results confirm the existence of response stereotypy in an MBON despite its random inputs: this analysis of locust *bLN1* is likely the second demonstration of response stereotypy following random connectivity in any nervous system, after the first one in flies by Hige et al.^20^. Our simulations show that stereotypy emerges within the total population of KCs, even though individual KCs do not show stereotypic responses. We also developed PRED, a new metric for quantifying stereotypy, and showed its advantages over the previously used correlation metric, especially for datasets which include responses to a small number of stimuli.

The simulations suggested that stereotypy does not require learning, and we confirmed this using an analytical model. The simulations instead pointed to other network properties that determine stereotypy. KCs receive their input from PNs, and the convergence of multiple KCs onto an MBON makes the MBON sensitive to fine differences in the PN responses to odors, such as a difference in total PN output. However, we also found that, even in the absence of differences in this total drive, stereotypy could arise from the differences in the total number of active PNs or their spiking ranges, with the help of the non-linearity in the transfer function of the KCs. Further, we showed with both simulations and experiments that stereotypy depends on the level of sparseness. The simulations also revealed that it is the total number of active KCs and not their spiking rate that is important for stereotypy. Finally, we showed that convergence:randomness ratio in a random network is an important determinant of stereotypy. Our simulations allowed us to assess the role of various network parameters in generating stereotypy (Figs. 3–6 and 8; Supplementary Figs. 2, 5, 7 and 8); the simulations also showed that stereotypy is robustly present even when the parameters are varied from the default values taken from flies, suggesting that our conclusions can be generalized to many species with different network parameters.

Although we confirmed stereotypy in MBON and total KC responses using in vivo data in locusts and flies, the observed levels of stereotypy were moderate and noisy across different pairs of odors and individuals. Some of this variability is likely a result of experimental noise, particularly in calcium imaging experiments. Physiological noise in the neurons may also reduce response stereotypy. Response stereotypy will contribute to behavioral stereotypy, which is perhaps preferable in moderation, as both behavioral consistency and behavioral stochasticity can have evolutionary benefits^52^.

Our results show that the existence of stereotypy does not require learning. Rather, learning experiences, occurring as individuals face different environments, may actually decrease the similarity in the neural and behavioral responses across individuals. This was indeed found to be the case in the mice accessory olfactory system^53^. Among insects, *rutabaga* mutant flies, which are deficient in learning, showed higher stereotypy^20^ across individuals than wild-type flies. Interestingly, the same study also showed that across-hemisphere stereotypy within a wild-type fly brain is higher than across-individual stereotypy^20^. One can speculate that the across-individual stereotypy may be as high as the across-hemisphere stereotypy in newborn flies, but while the former reduces over time due to the different experiences of individuals, the latter is maintained as the two hemispheres share the experiences. In flies, dopaminergic neurons, which send reinforcement signals to the mushroom body^43,54–57^ and some of which have bilateral projections^19,58^, may contribute to maintaining across-hemisphere stereotypy.

The default network in our simulations included the assumption that PN responses are fully stereotyped and the PN-KC connections are fully random. While these assumptions reflect the prevalent view in the field^8–10,14–18^, there are counter reports as well. A recent study analyzing the fine structure of receptor neuron to PN synapses reported some variability in the synapses^59^, although the functional impact of this variability on PN response stereotypy remains to be understood. Another factor that could add variability to PN responses is the plasticity within the antennal lobe^60,61^. PN-KC connectivity patterns are also debated. Some studies reported that PN axonal projections to the mushroom body are stereotyped but KC dendritic projections are not^18,62–64^, while others found that both are not stereotyped^8,36,65^. Despite this uncertainty, both kinds of studies imply that the fine connections between PNs and KCs are random, although some coarse-level biases in connectivity have not been ruled out^17,65^. It is also possible that some KCs receive non-random inputs^66^. Our results show that in a circuit with only partial randomness in connectivity, stereotypy can be achieved even without massive amounts of convergence (Fig. 8b, c). In the competition between randomness and convergence, the convergence:randomness ratio appears to be the determining factor for stereotypy. Partial randomness and partial stereotypy in PN-KC connections could also provide a way for different MBONs to have reliable differences in their information content, in addition to having different levels of convergence^19^ and different temporal patterns^29,42^; MBONs also differ in the areas they project to and the modulatory inputs they receive^19,67^.

Individual-to-individual variation has been previously studied in invertebrate central pattern generators like the lobster stomatogastric ganglion wherein the phases of generated rhythms maintain remarkable stereotypy despite differences in the membrane conductance of neurons or synaptic strengths between coordinating partners across individuals^68,69^. However, unlike the largely feedforward PN-KC-MBON circuit (although some feedback has been observed^19,40,66^), the stomatogastric circuits are recurrent networks with significant feedback loops^68^. The two systems differ also in that connections between neurons in central pattern generators are mostly invariant, whereas in the mushroom body an entire neural layer is randomly connected to its predecessor.

Experimental studies have shown that KC responses are sparse such that only about 5–10% of KCs respond to any odor^39,49,50^. Sparse representations have been proposed to be particularly suitable for learning and memory^70^. If so, wouldn’t it be better to have an even smaller fraction of KCs respond to any odor? One likely reason for not having more sparseness is that the observed level of sparseness may provide a good trade-off between the discrimination and generalization of odors^71^. Our results provide another possible reason: more sparseness would be detrimental for response stereotypy (Figs. 6 and 7). Interestingly, our simulations revealed a non-uniform increase in stereotypy with the fraction of active KCs: stereotypy increased until the proportion of active KCs reached 10–15%, and saturated beyond that. It is therefore tempting to speculate that the sparseness of KCs may have been tuned by evolution to strike a balance between response stereotypy and efficient learning or generalization. Given that stereotypy is influenced by the absolute number of active KCs, changing the total number of KCs during evolution while keeping sparseness (i.e., fraction) the same could be another way to alter response stereotypy.

We did not explicitly include inhibitory neurons in the model, but their effects were taken into account indirectly. In the antennal lobe, inhibitory local neurons shape the responses of PNs—this inhibition was accounted for when we set the firing rates of PNs in the model to match the known firing rates. The sparseness of KCs is maintained by a GABAergic inhibitory neuron^51,72^. The inhibitory effect was accounted for by setting the spiking threshold of KCs to match the experimentally observed responses of KCs. The direct KC-MBON synapses have been observed to be mostly excitatory^20^, although there may be indirect inhibitory effects due to lateral connections among MBONs^42^.

KCs are among the largest neuronal populations in the insect brain^32^. Precise genetic specification of synapses between individual neurons in large populations may be inefficient or impossible. Our results suggest that it may even be unnecessary, as convergence following the random connectivity allows reliable extraction of sensory information. Although it has only been possible to evaluate the randomness of connectivity in a few model systems^7,8,26^, it is likely to be a more common motif in bigger brains containing larger populations of neurons. Individual neurons in randomly connected networks may respond differently, yet at the level of neurons receiving densely converging inputs, your brain probably generates the same neural activity in response to a red stimulus as my brain does.

## Methods

### PRED stereotypy metric

To estimate pairwise relative distance (PRED) stereotypy, we observed the neuronal responses in a pair of individuals (say, A and B) for a pair of odors (say, 1 and 2). *D*_1_ was defined as the squared difference between the total response to the same odor in two individuals, and *D*_2_ was defined as the squared difference between the total response to different odors in two individuals (Fig. 1c). Stereotypy was quantified as $\frac{{D_2 - D_1}}{{D_2 + D_1}}$ and varied between −1 and 1 (Fig. 1c). If the responses are similar across individuals but vary with the odor, *D*_1_ would be close to 0 and *D*_2_ would be large, giving a positive value for PRED stereotypy. If the responses of two individuals differ as much for same odors as they do for different odors (or if the responses are random), *D*_1_ on average would be equal to *D*_2_, giving PRED stereotypy values close to 0. PRED stereotypy is negative if the odor generating the higher response in one individual generates the lower response in the other individual, and vice versa. If all responses were equal, the value of PRED stereotypy was set to 0. If the number of odors or individuals was more than two, PRED stereotypy was calculated for all possible pairs and then averaged.

### Locust intracellular recordings

We obtained sharp intracellular recordings from an earlier study^29^, which had collected odor responses of various MBONs in locusts but had not looked at stereotypy. These recordings were made in vivo from awake animals in the *β*-lobe of the locust mushroom body. In this dataset, the cell-type was identified based on the recording location and the response characteristics, and in most cases confirmed with dye-fills. We focused on the class *bLN1*, which has only one neuron per mushroom body and shows dense dendritic projections in the *β*-lobe^29^. We analyzed a set of recordings of the *bLN1* neuron in 6 different individuals, all of which were tested for a set of 6 odor stimuli (0.1 and 10% concentrations of cyclohexanone, octanol, and hexanol each; see Fig. 1a and Supplementary Fig. 1a and b). The response was quantified as the number of spikes observed in a 2-s response period following odor onset, minus the number of spikes in a 2-s period before the stimulus (averaged over 10 trials). Although the odor was presented for 1 s only, we used a 2-s window as the responses often lasted longer than the stimulus duration.

### *Drosophila* PN and KC datasets

We obtained whole-cell patch-clamp PN recordings from an earlier study^73^ for four classes of PNs: VC4, DL2v, VM5v, and VC3; for each class, responses to 2–4 odors were available in 2–6 individuals. The trial-averaged response was quantified as described for locust *bLN1* recordings. The KC responses were extracted from Fig. 3a of Murthy et al.^16^ containing whole-cell patch-clamp recordings from a single clonal population (left lateral posterior clonal unit) of KCs. Only binary responses could be extracted, but they are a reasonable approximation for the highly sparse KC responses. KCs belonging to the same class were treated as the same for the calculation of stereotypy.

### Simulations

We simulated the responses and inputs of an MBON using a network of rectified linear units. The network consisted of 50 PNs^8,30,31^, 2000 KCs^32^, and the MBON (Fig. 2a). Connections between PNs and KCs were modeled as a random binary matrix, where 1 or 0 denote the presence or absence of a connection, respectively. The connection probability, i.e., the fraction of 1s in the matrix, was set to 0.14 so that each KC, on average, was connected to 7 PNs^8,74^. The MBON received input from a fixed subset of 1000 KCs^40^, set to the first 1000 KCs without loss of generality. PN-KC connection matrix was generated randomly for each individual in a simulation, while the KC-MBON connections were identical in all individuals.

For any given odor, each PN responded with 0.5 probability^15,38^. For responding PNs, the number of spikes was drawn from a uniform distribution in the range of 10–30 (mean 20), corresponding to a brief response window^15,38^. For non-responding PNs, the number of spikes was set to 0. For the KC and MBON layers, we used the standard rectifier function to calculate the response of a unit, *f*(*x*) = max[0,*k* − *t*], where *k* denotes the total input received by the unit and *t* denotes the response threshold. The value of *t* was set such that ~10% KCs responded to odor presentations^38,39^; the same value (*t* = 119) was used in all simulations, except when we tested the effect of the threshold on stereotypy. Note that PN response vectors varied with the odor but not with the individual, while PN-KC connection matrices varied with the individual but not with the odor. Each network simulation was performed 100 times with different initializations of the random number generator.

### Reanalysis of the effect of learning

We used the code from the GitHub repository (commit code: c050be6) provided by Schaffer et al.^24^. We ran the scripts calculateSumFigParts.m and makeFigure2.m without any modifications and noted the stereotypy in the piriform cortex responses for the three odor sets as defined by the researchers^24^; in their approach, the stereotypy was estimated using the correlation coefficient between two response vectors (one for each cortex), where each vector contained the responses to a panel of odors. We also calculated the stereotypy using our distance-based method, by first calculating the value for each pair of odors and then taking the average. For removing weight normalization we ran the same code after commenting lines 162–163 in calculateSumFigParts.m. Because the random number generator was not initialized in the provided code, the simulations produced slightly different outputs on every run. To increase confidence in the results, we repeated the simulations six times.

### Analytical model

The analytical model was based on the framework provided by Jortner^75^, using binary responses for PNs and KCs, and a binary matrix to represent the PN-KC connections. A KC is considered to have a response if its net input crosses a threshold. We derive closed-form expressions for *D*_1_ and *D*_2_, which allow estimation of stereotypy without numerical simulations (see Supplementary equations).

### Simulations with fixed input drives

To equalize the input drives from the PN population to the KC population for different odors, we first fixed the total number of spikes in the PN layer to 500 (mean spiking rate times half the number of PNs) and then distributed them randomly among the active PNs. In simulations where we varied the spiking range of PNs, we set the number of active PNs to be exactly 25, half the total number of PNs. Then we set the spike rate of these 25 PNs by randomly partitioning the set of 500 spikes into 25 discrete subsets while ensuring that the size of each subset was within the desired range of PN spiking. In simulations where we varied the number of active PNs, the set of 500 spikes was partitioned among the chosen number of active PNs while ensuring that the number of spikes in each active PN was within the default spiking range of 10–30.

### *Drosophila* calcium imaging

Flies were imaged using two-photon laser scanning microscopy using the protocol described earlier^51^. Briefly, the cuticle and trachea overlying the mushroom bodies were removed and the brain was superfused with artificial hemolymph (5 mM TES, 103 mM NaCl, 3 mM KCl, 1.5 mM CaCl_2_, 4 mM MgCl_2_, 26 mM NaHCO_3_, 1 mM NaH_2_PO_4_, 8 mM trehalose, and 10 mM glucose, pH 7.3) bubbled with carbogen (95% O_2_, 5% CO_2_). 5-s pulses of isoamyl acetate, ethyl acetate, or delta-decalactone (Sigma) were delivered by switching mass-flow controlled carrier and stimulus streams (Sensirion) via software-controlled solenoid valves (The Lee Company). Flow rates at the exit port of the odor tube were 0.5 ml min^−1^.

Fluorescence was excited by ~70 fs pulses of 910 nm light from a Mai Tai eHP DS Ti-Sapphire laser (Spectra-Physics), attenuated by a Pockels cell (Conoptics) and coupled to Movable Objective Microscope (Sutter Instrument Co.) with a galvo-resonant scanner. Excitation light was focussed by a 20 × 1.0 NA objective (Olympus XLUMPLFLN20XW) and emitted light was detected by GaAsP photomultiplier tubes (Hamamatsu Photonics, H10770PA-40SEL) and amplified by a TIA-60 amplifier (Thorlabs). Volume imaging was enabled by a piezo objective stage (nPoint, nPFocus400) and the microscope was controlled by ScanImage 5 (Vidrio).

Movies were motion-corrected in X-Y using the moco ImageJ plugin^76^, with pre-processing to collapse volume movies in Z and to smooth the image with a Gaussian filter (standard deviation = 4 pixels; the displacements generated from the smoothed movie were then applied to the original, unsmoothed movie), and motion-corrected in Z by maximizing the pixel-by-pixel correlation between each volume and the average volume across time points. ∆F/F was calculated as earlier^51^.

### *Drosophila* genetics and structural imaging

Tetanus toxin (TNT) was expressed stochastically in APL via the intersection of NP2631-GAL4 and GH146-FLP, as described earlier^51^. The full genotype was *NP2631-GAL4, GH146-FLP/tub-FRT-GAL80-FRT, UAS-TNT, tubP-GAL80*^*ts*^; *mb247-LexA, lexAop-GCaMP3/UAS-mCherry* or *NP2631-GAL4, GH146-FLP/UAS-TNT, tubP-GAL80*^*ts*^; *mb247-LexA, lexAop-GCaM3P/tub-FRT-GAL80-FRT*. Flies were raised at 18 °C and heated to 31 °C for 16–24 h before the experiment to acutely induce TNT expression in APL. In flies with *UAS-mCherry*, TNT expression in APL was scored in unfixed, dissected brains by whether APL expressed mCherry. In flies without *UAS-mCherry*, TNT expression was scored by anti-TNT immunohistochemistry following an existing protocol^77^. Briefly, dissected brains were fixed in 4% (w/v) paraformaldehyde in PBT (100 mM Na_2_HPO_4_/NaH_2_PO_4_, pH 7.2 with 0.3% (v/v) Triton X-100) for 20 min at room temperature. Samples were washed in PBT 2x quickly then 3 × 10 min, blocked in PBT + 5% goat serum for 30 min, incubated in 1:100 rabbit anti-TNT antibody (Abcam, ab53829) for 2 d at 4 °C, washed in PBT 2x quickly then 3 × 10 min, incubated in 1:1000 Alexa 546-conjugated goat anti-rabbit secondary (Thermo Fisher, A-11071), washed in PBT 2x quickly then 3 × 10 min, and mounted in Vectashield (Vector Labs, H-1000).

### Stereotypy versus convergence:randomness ratio

In simulations where both the randomness in PN-KC connections and the convergence (the KC-MBON connection probability) were varied, we used the MATLAB curve-fitting toolbox to fit the stereotypy values to the ratio of convergence to randomness (Fig. 8c). As the dependence showed a sigmoidal shape, we fitted the data using the standard Hill equation

$$S = \frac{{r^a}}{{b + r^a}}$$

with the ratio (*r*) as the independent variable and stereotypy (*S*) as the dependent variable. The values of the parameters *a* and *b* were estimated by non-linear least squares regression using the *fit* function, and the goodness of fit was determined using the coefficient of determination (*R*^2^).

### Statistics

Unless specified otherwise, *n* = 100 in simulations, denoting the total number of network iterations with random seeds. Comparisons of stereotypy with baseline (0) were made using one sample two-tailed *t*-test, except otherwise specified. Correlations were quantified using Pearson’s correlation coefficient.

### Reporting summary

Further information on research design is available in the Nature Research Reporting Summary linked to this article.

## Supplementary information

Supplementary Information

Reporting Summary

## Data Availability

All data for simulations can be regenerated using the code provided (see Code availability). The processed files for data from other sources used in the study are also available in the code repository. A reporting summary for this Article is available as a Supplementary Information file.

## Code availability

The simulations were coded in MATLAB (version r2016a, The MathWorks Inc., Natick, USA). The figures were plotted using a modified version of the gramm plotting package^78^. All source code is available at https://github.com/neuralsystems/magnitude_stereotypy.
