# Supplementary material for: Multiple network properties overcome random connectivity to enable stereotypic sensory responses
Source: Nat Commun. 2020 Feb 24;11:1023. doi: 10.1038/s41467-020-14836-6 (PMC7039968; doi:10.1038/s41467-020-14836-6)
Supplement: Supplementary file 1 — Supplementary Information [file 41467_2020_14836_MOESM1_ESM.pdf]

Supplementary Information

Multiple network properties overcome random connectivity to enable stereotypic sensory responses

Mittal et al.

Supplementary Figure 1

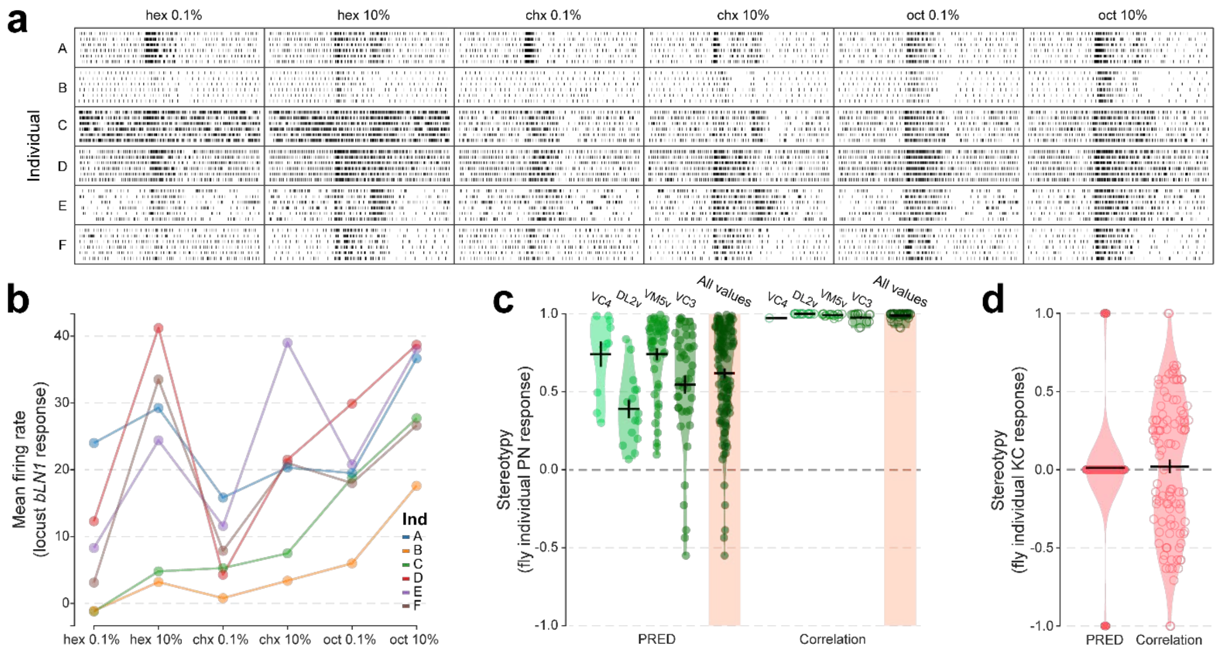

Supplementary Figure 1: Experimental data from locusts and *Drosophila*.

a Raster plots showing the response of locust *bLN1* to all six odor stimuli in six different individuals. Each experiment had between 6 and 11 repeated trials, of which the first 6 are displayed. **b** Summary of the response magnitudes extracted from the *bLN1* recordings in **(a)**. Although the responses were varied across individuals, some similarities could be noted; for example, octanol (oct) typically generated stronger responses than cyclohexanone (chx) for either concentration. **c** PRED and correlation stereotypy values calculated from electrophysiological data for four classes of PNs in *Drosophila* (see **Methods**). **d** PRED and correlation stereotypy values calculated from electrophysiological data for genetically labeled KCs in *Drosophila* (see **Methods**).

Supplementary Figure 2

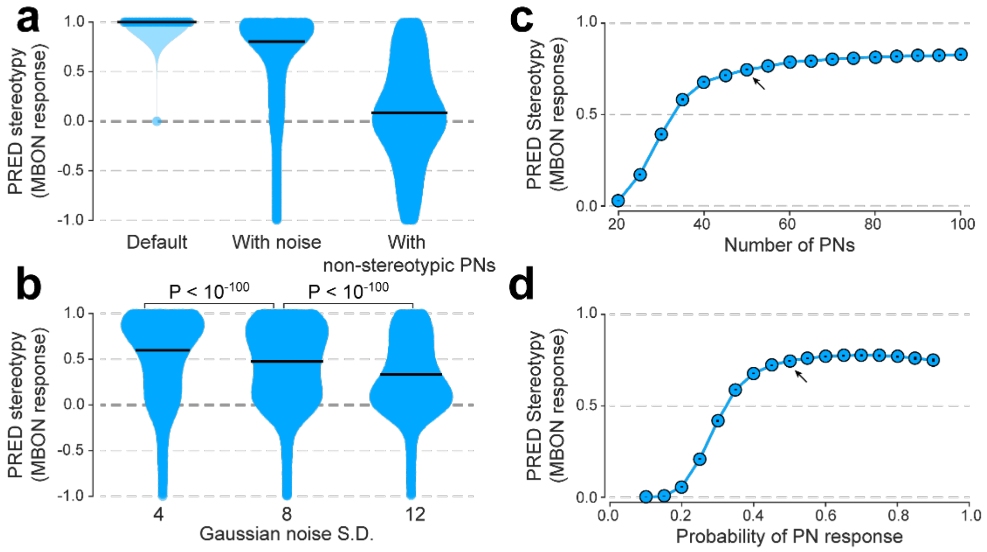

Supplementary Figure 2: Stereotypy in MBON response for different variations of the default network.

a Stereotypy in MBON response in a (dummy) network with identical connections between PNs and KCs across individuals. As expected**,** there is perfect stereotypy in this simulation **(left panel)**. Stereotypy reduces only slightly if noise **(middle panel)** is added to all neurons; noise term is drawn from a Gaussian distribution with mean equal to 0 and standard deviation equal to 2 (this noise was relatively small compared to the variability observed in PN recordings). Stereotypy in the MBON is lost completely **(right panel)** if the odors generate independent PN responses across individuals (i.e., if there is no stereotypy in PN responses). **b** Stereotypy in MBON response reduces with increase in the amount of Gaussian noise added to all the neurons in the simulations of a real network as illustrated in **Fig. 2a**. The noise was drawn from a Gaussian distribution with 0 mean and the indicated standard deviation. P-values from t-tests are shown in the figure. **c, d** Stereotypy in MBON response versus the number of PNs in the network **(c)** or the probability of PN response to an odor **(d)**. The simulations are done with a realistic network with random PN-KC connections. The responses are calculated for 100 odors in each simulation. The default number of PNs was 50 and the default probability of PN response was 0.5 (indicated by arrows). In panels **(a)** and **(b)**, n = 495000 points corresponding to 100 iterations × 4950 odor pairs from 100 odors. In panels **(c)** and **(d)**, n = 100 iterations. In all panels, error bars represent s.e.m.

Supplementary Figure 3

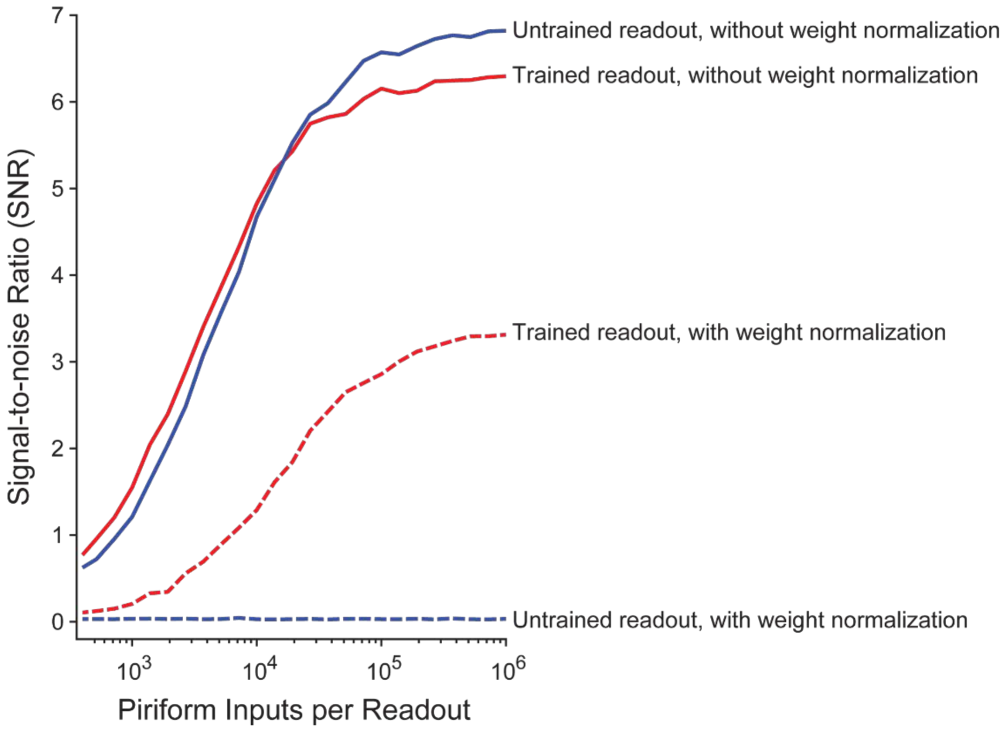

Supplementary Figure 3: Signal-to-noise ratio in simulations without weight normalization.

The plots compare the signal-to-noise ratio (SNR) in the simulations with and without weight normalization, using code provided by Schaffer et al.[1]. The SNR was higher without the weight normalization, in both the trained and the untrained networks.

Supplementary Figure 4

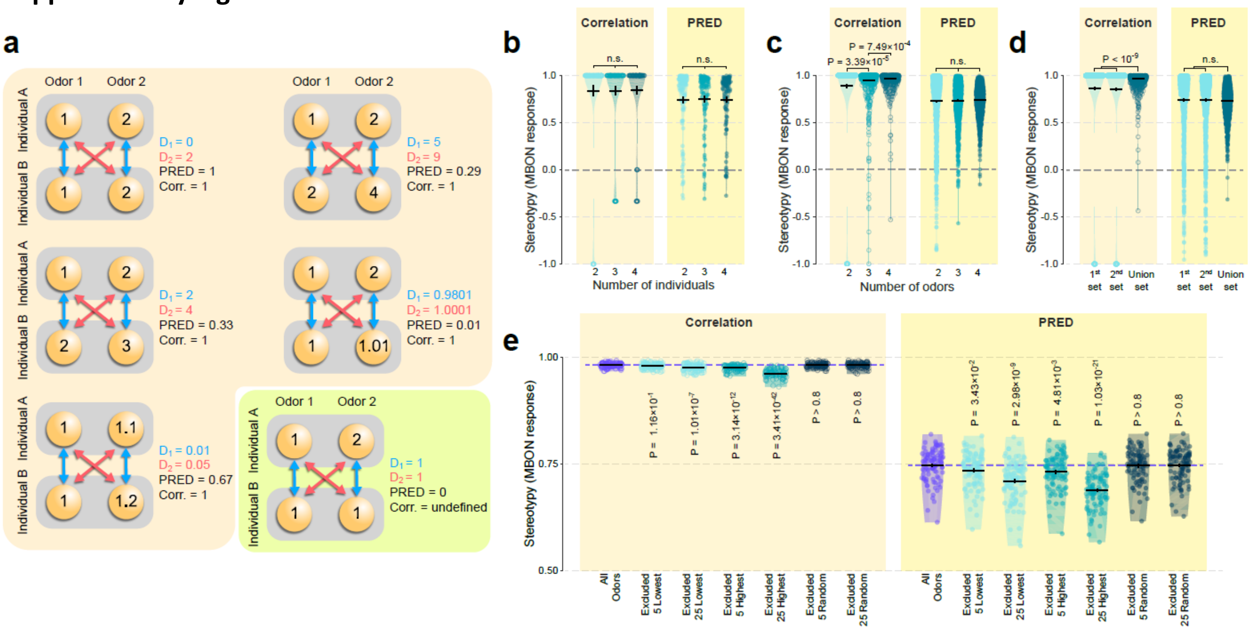

Supplementary Figure 4: Comparison of correlation and PRED metrics for stereotypy

a PRED is a more sensitive metric for stereotypy. In all of the first five examples, the correlation stereotypy is exactly 1. The PRED stereotypy, on the other hand, takes different values depending on the absolute differences between the neural responses for both odors across the individuals. In the last example, the correlation stereotypy is undefined but the PRED stereotypy is appropriately 0 (note that in this example, it is not possible to accurately predict the odor identity from a response in one individual if the responses in the other individual are known). **b** Stereotypy as a function of the number of individuals, in simulations with 2 odors. Both metrics gave unvarying estimates of stereotypy as the number of individuals was varied; $n = 100$ network iterations. **c** Stereotypy as a function of the number of odors. The specified number of odors (2, 3, or 4) were randomly sampled for calculating the stereotypy values from a simulation with 100 odors. The correlation metric increased with the number of odors while the PRED metric did not; $n = 1000$ random samplings of the specified numbers of odors out of the total 100 odors. **d** In the same simulation as in **(c)**, two non-overlapping sets of 2 odors each were selected randomly for calculating the stereotypy values and expectedly showed equal stereotypy by either metric. Next, the two sets were combined and stereotypy was calculated for the union set of 4 odors. The correlation stereotypy was greater for the union set as compared to the individual sets, while the PRED stereotypy did not change; $n = 1000$ repetitions of the samplings and stereotypy calculations. **e** In simulations with 100 odors, we ranked the odors according to how many PNs they activated, and then calculated correlation and PRED stereotypy for the full set of odors or for reduced sets by excluding specific odors. Stereotypy reduced slightly when 5 or 25 odors with the lowest activity were removed, or when 5 or 25 odors with the highest activity were removed, but not when 5 or 25 random odors were removed. Statistical comparisons shown are with respect to the "all odors" group for each metric; $n = 100$ network iterations. In panels **(b)—(e)**, t-tests were used for comparisons. Non-significant differences are indicated as n.s. Error bars represent s.e.m.

Supplementary Figure 5

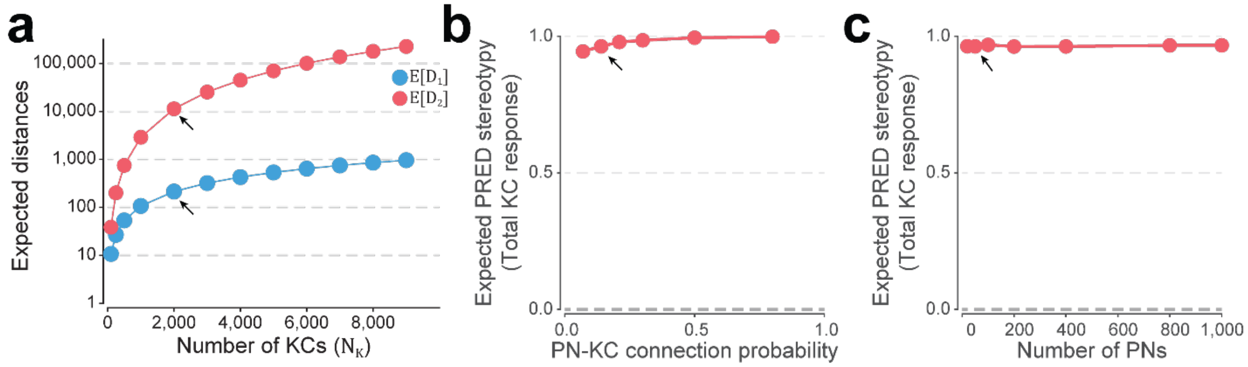

Supplementary Figure 5: Calculations with the analytical model.

a $E[D_1]$ and $E[D_2]$ in the analytical model. The values were calculated using the formulae derived in the analytical model, for different numbers of KCs, and are shown on a log scale. **b** Expected PRED stereotypy remains high for different values of PN-KC connection probability. **c** Expected PRED stereotypy remains high for different values of the number of PNs. In all calculations, the KC threshold was adjusted to maintain their spiking probability at 0.1. Arrows indicate the default values (taken from the *Drosophila* system).

Supplementary Figure 6

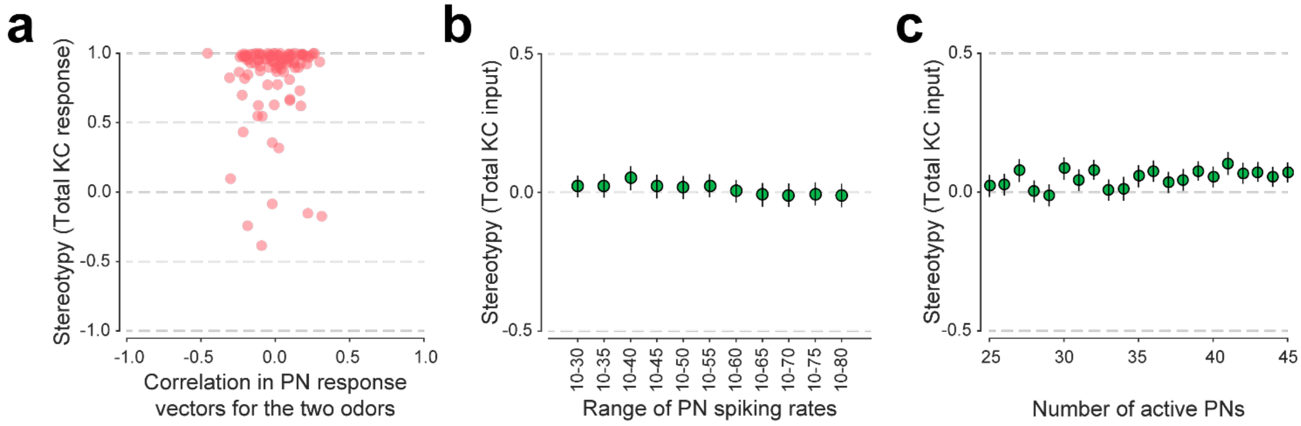

Supplementary Figure 6: Stereotypy as a function of PN response features.

a Scatter plot of stereotypy in total KC response versus the correlation between the PN response vectors for the two odors in each iteration. Pearson's correlation coefficient between the two quantities is 0.03 ($P = 0.78$), showing that the stereotypy in total KC response does not depend on the correlations among the PN response vectors. **b, c** There is no stereotypy in total KC input when input drives to KCs are fixed. Stereotypy in total KC input versus the range of PN spiking rates **(b)** in the same simulations as in **Fig. 5c,** and versus the number of active PNs **(c)** in the same simulations as in **Fig. 5d**. In all panels, n = 100 iterations; error bars represent s.e.m.

Supplementary Figure 7

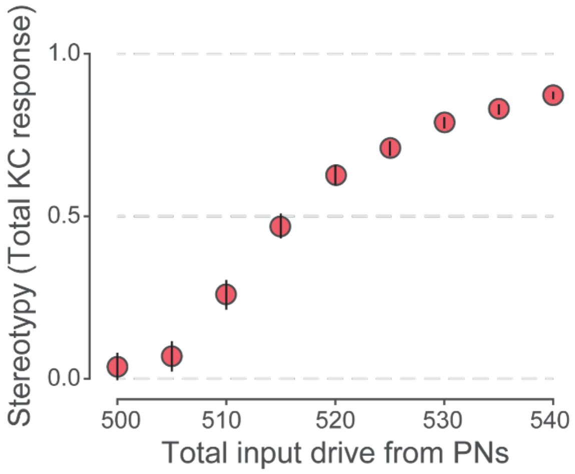

Supplementary Figure 7: Stereotypy versus total input drive when the total number of active PNs and their spiking rate range is the same for the two odors.

In these simulations, the total input drive is changed to the indicated values for one odor while it is maintained at 500 for the other; n = 100 iterations. Error bars represent s.e.m.

Supplementary Figure 8

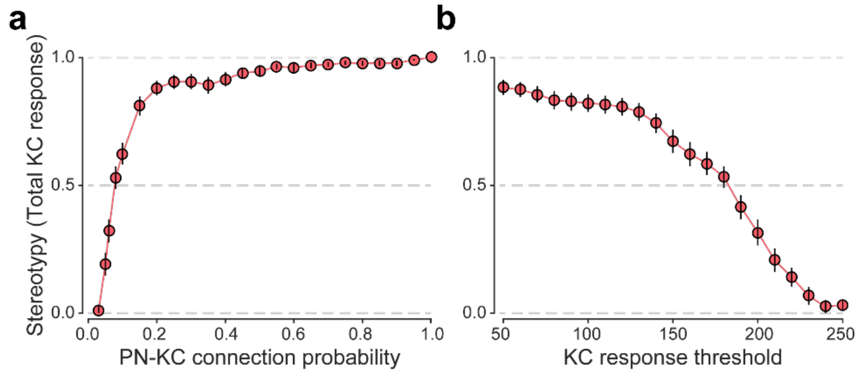

Supplementary Figure 8: Stereotypy increases with a general increase in inputs to KCs.

a Stereotypy in total KC response versus the PN-KC connection probability in our simulations. **b** Stereotypy versus the KC response threshold; note that lowering the threshold has the same effect as increasing the input. In all panels, n = 100 iterations; error bars represent s.e.m.

Supplementary Figure 9

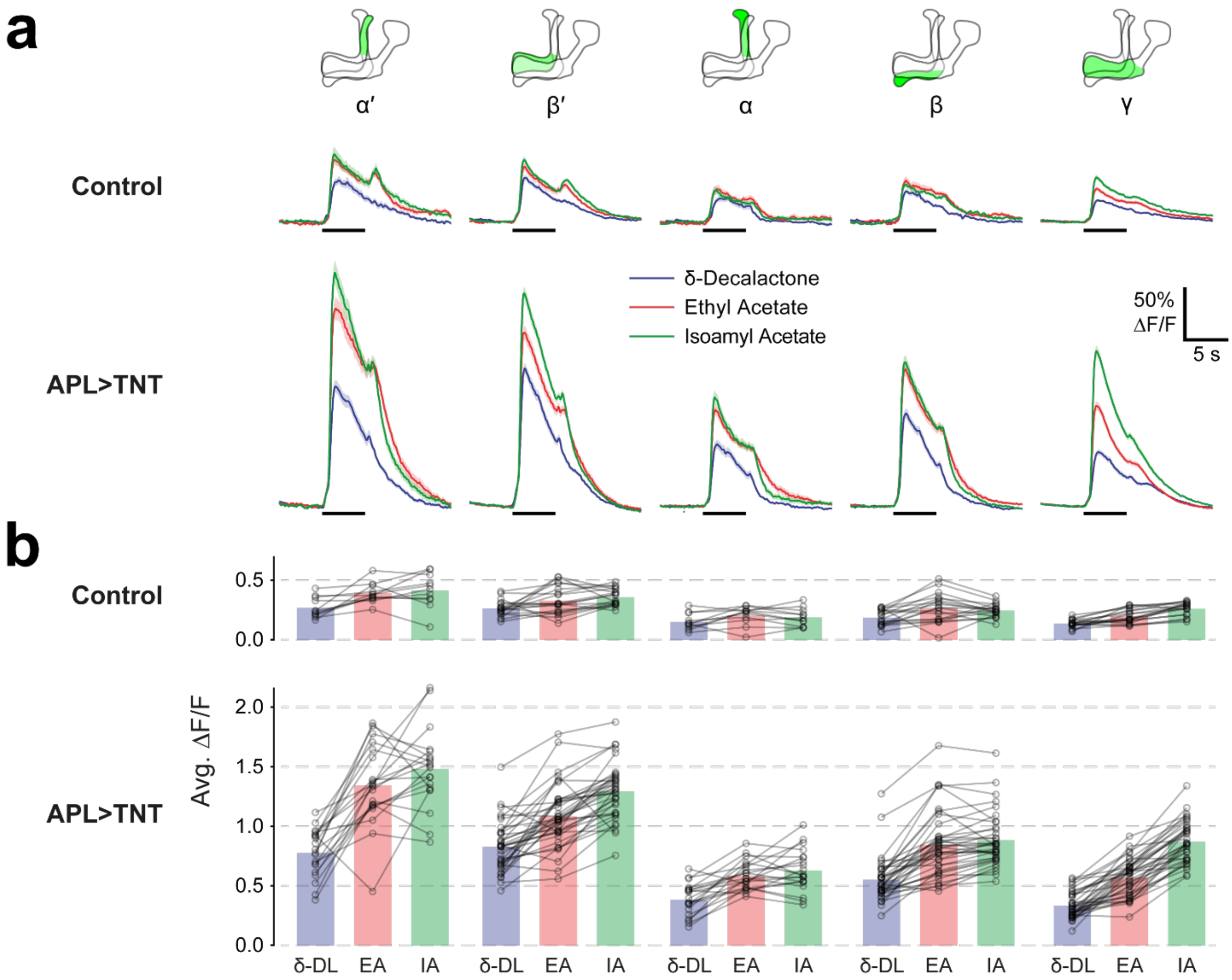

Supplementary Figure 9: Raw data used to calculate stereotypy in Fig. 7d

a ΔF/F traces of odor responses in the α′, β′, α, β, and γ lobes of the mushroom body (shown in diagrams above), in flies expressing GCaMP3 in Kenyon cells (*mb247-LexA>GCaMP3*) and stochastic expression of tetanus toxin (TNT) in the APL neuron (see Methods). Top row, control: hemispheres where APL did not express TNT. Bottom row, APL>TNT: hemispheres where APL did express TNT. Shading on traces indicates s.e.m. Black bars indicate 5-s odor pulses. Blue, δ-decalactone (δ-DL); red, ethyl acetate (EA); green, isoamyl acetate (IA). Scale bars: 50% ΔF/F (vertical); 5 s (horizontal). **b** Mean ΔF/F during the 5 s of the odor pulse for data shown in **(a)**. Labels and colors as in **(a)**. Individual data points and connecting lines indicate single brain hemispheres imaged. n given as number of hemispheres (number of flies): α′ and α: control, 11 (10), APL>TNT, 20 (14). β′, β, and γ: control, 18 (15); APL>TNT, 36 (24).

Supplementary equations

Mushroom body neural network

We start our theoretical calculations using a framework similar to the one previously described by Jortner[2], which we then expand to explicitly calculate the outputs of Kenyon cells and stereotypy in the total KC response. We define the responses of projection neurons (PN) to an odor as a binary vector ($\vec{P}$) of length equal to the number of PNs (N_P),

$$\vec{P} \in \{0,1\}^{N_P \times 1}$$

Here, $P_i = 1$ with probability p and denotes that the ith PN responds to the odor; $P_i = 0$ denotes no response. Similarly, we define the responses of Kenyon cells (KC) as a binary vector ($\vec{K}$) of length equal to the number of KCs (N_K),

$$\vec{K} \in \{0,1\}^{N_K \times 1}$$

We define the connections between the PN and KC layers using a random binary matrix ($\overleftrightarrow{M}$) with fixed weights (1 or 0),

$$\overleftrightarrow{M} \in \{0,1\}^{N_K \times N_P}$$

The ith row of this matrix ($\overrightarrow{M_i}$) tells which PNs are connected to the ith KC. $M_{ij} = 1$ with probability c and denotes that the jth PN is connected to the ith KC; $M_{ij} = 0$ denotes no connection. Each KC integrates the input received from each of the PNs connected to it. The input to the ith KC from the jth PN is given by $M_{ij}P_j$. Thus, the total input to the ith KC (k_i) from all the PNs is given by

$$k_i = \sum_{j=1}^{N_P} M_{ij} P_j$$

The value of k_i can be an integer between 0 and N_P. Each KC responds only if the total input it receives is above a threshold (t). The response of the ith KC (K_i) is computed as

$$K_i = \begin{cases} 1 & \text{for } k_i \geq t \\ 0 & \text{for } k_i < t \end{cases}$$

KC response threshold

Since $\overrightarrow{M_i}$ and $\vec{P}$ are both binary vectors, $M_{ij}P_j$ is also binary and is 1 only when both M_{ij} and P_j are 1. Because the two terms are independent with probabilities c and p, respectively,

$$\begin{aligned} \Pr[M_{ij}P_j = 1] &= pc \\ \Pr[M_{ij}P_j = 0] &= 1 - pc \end{aligned}$$

As all PNs and connection vectors are independent of each other, calculating the input to the ith KC is akin to counting the number of 1s resulting from N_p independent experiments in which the probability of getting a 1 is pc. Therefore, k_i follows a binomial distribution,

$$k_i \sim \mathbf{B}(N_P, pc)$$

The probability of getting a particular input to the KC is given by the probability density function of the binomial distribution such that

$$\Pr[k_i = r] = \binom{N_P}{r}(pc)^r(1-pc)^{N_P-r},\ r \in [0, N_P]$$

The probability that the input is less than the threshold is given by

$$\Pr[k_i < t] = \sum_{r=0}^{t-1}\binom{N_P}{r}(pc)^r(1-pc)^{N_P-r}$$

When $k_i < t$, the ith KC does not respond. If the desired response probability of KCs is q, the value of t should be set such that $\Pr[k_i < t]$ is equal to $1-q$. Because the binomial distribution is discrete and no value of t may give the exact probability, we define our threshold as the maximum value (x) that satisfies the condition $\Pr[k_i < x] \le 1-q$. Putting together all the equations, we have

$$t = max(x)\ \left|\ \sum_{r=0}^{x-1}\binom{N_p}{r}(pc)^r(1-pc)^{N_P-r} \le 1-q\right.$$

Total KC response

The total KC response (κ) is defined as

$$\kappa = \sum_{i=1}^{N_K} K_i$$

κ takes integer values between 0 and N_K. Next, we calculate the probability that $\kappa = r$ for any $r \in [0, N_K]$. The response of every KC depends on the number of PNs that are active for a given odor. We denote the number of active PNs as $\mathbf{ones}(\vec{P})$; by marginalization over all possible values of this parameter, we calculate

$$\Pr[\kappa = r] = \sum_{v=0}^{N_P}\Pr\left[\kappa = r \mid \mathbf{ones}(\vec{P}) = v\right]\cdot\Pr\left[\mathbf{ones}(\vec{P}) = v\right]$$

Since $\vec{P}$ is a binary vector, the probability of having v $1s$ in $\vec{P}$ is given by the binomial probability distribution function,

$$\begin{aligned}\Pr\left[\mathbf{ones}(\vec{P}) = v\right] &= \binom{N_P}{v}p^v(1-p)^{N_P-v}\\ &\stackrel{\text{def}}{=} \mathbf{pdf}(v; N_P, p)\end{aligned}$$

To spike, a KC needs to get input from at least t of the v responding PNs. The probability that it gets input from any PN is c, as described earlier. Therefore,

$$\begin{aligned}\Pr\left[K_i = 1 \mid \mathbf{ones}(\vec{P}) = v\right] &= \sum_{s=t}^{v}\binom{v}{s}c^s(1-c)^{v-s}\\ &\stackrel{\text{def}}{=} \mathbf{ccdf}(t; v, c)\end{aligned}$$

Henceforth, we use the functions $\mathbf{pdf}$ and $\mathbf{ccdf}$ as defined in the two equations above. Since all KCs get input from the same PN vector they are not independent of each other. KC responses depend on the

number of $1s$ in $\vec{P}$. If the number of $1s$ in $\vec{P}$ is constrained, KC responses do not depend any further on $\vec{P}$ and become independent of each other. Thus, given the constraint $\mathbf{ones}(\vec{P}) = v$, K_i follows the binomial distribution $\mathbf{B}(N_K, \mathbf{ccdf}(t; v, c))$. Therefore,

$$\Pr\left[\kappa = r \mid \mathbf{ones}(\vec{P}) = v\right] = \mathbf{pdf}(r; N_K, \mathbf{ccdf}(t; v, c))$$

Finally,

$$\Pr[\kappa = r] = \sum_{v=0}^{N_P} \mathbf{pdf}\,(r; N_K, \mathbf{ccdf}(t; v, c)) \cdot \mathbf{pdf}(v; N_P, p)$$

Calculation of stereotypy

Now we extend the notation to refer to different individuals and odors. $\overleftrightarrow{M^{(I)}}$ denotes the connectivity matrix for individual I (connectivity matrix does not depend on the odor). $\overrightarrow{P^{(O)}}$ denotes the PN response vector for odor O (PN vector does not depend on the individual). $\kappa^{(I,O)}$ denotes the total KC response of individual I to odor O. We calculate stereotypy (S) for the case of two individuals and two odors,

$$S = \frac{D_2 - D_1}{D_2 + D_1}$$

where, D_1 denotes the squared-distance between total KC responses of two different individuals to an odor, and D_2 denotes the squared-distance between total KC responses of two different individuals for two different odors (**Fig. 1C**). The expected value of stereotypy in the system can be calculated as

$$E[S] = E\left[\frac{D_2 - D_1}{D_2 + D_1}\right]$$

The first order approximation of the term is

$$\begin{aligned} E[S] &= \frac{E[D_2 - D_1]}{E[D_2 + D_1]} \\ &= \frac{E[D_2] - E[D_1]}{E[D_2] + E[D_1]} \end{aligned}$$

In the next sections we calculate the values of $E[D_1]$ and $E[D_2]$.

Expected distance between total KC responses in two different individuals for the same odor ($\boldsymbol{E[D_1]}$)

For two individuals (say, A and B) responding to an odor O, D_1 equals $(\kappa^{(A,O)} - \kappa^{(B,O)})^2$. The expected value of the term is

$$E[D_1] = \sum_{r_1=0}^{N_K} \sum_{r_2=0}^{N_K} (r_2 - r_1)^2 \cdot \Pr\left[(\kappa^{(A,O)} = r_1) \cap (\kappa^{(B,O)} = r_2)\right]$$

By marginalization over the number of $1s$ in $\overrightarrow{P^{(O)}}$,

$$\Pr\left[\left(\kappa^{(A,O)} = r_1\right) \cap \left(\kappa^{(B,O)} = r_2\right)\right] = \sum_{v=0}^{N_P} \left(\Pr\left[\left(\kappa^{(A,O)} = r_1\right) \cap \left(\kappa^{(B,O)} = r_2\right) \mid \mathbf{ones}(\overrightarrow{P^{(O)}}) = v\right] \cdot \Pr\left[\mathbf{ones}(\overrightarrow{P^{(O)}}) = v\right]\right)$$

Again, by constraining the number of $1s$ in $\overrightarrow{P^{(O)}}$, the total responses of KCs in the two individuals become independent. Therefore,

$$\begin{aligned}\Pr\left[\left(\kappa^{(A,O)} = r_1\right) \cap \left(\kappa^{(B,O)} = r_2\right) \mid \mathbf{ones}(\overrightarrow{P^{(O)}}) = v\right] &= \Pr\left[\kappa^{(A,O)} = r_1 \mid \mathbf{ones}(\overrightarrow{P^{(O)}}) = v\right] \cdot \Pr\left[\kappa^{(B,O)} = r_2 \mid \mathbf{ones}(\overrightarrow{P^{(O)}}) = v\right] \\ &= \mathbf{pdf}(r_1; N_K, \mathbf{ccdf}(t; v, c)) \cdot \mathbf{pdf}(r_2; N_K, \mathbf{ccdf}(t; v, c))\end{aligned}$$

Putting it all together,

$$E[D_1] = \sum_{r_1=0}^{N_K} \sum_{r_2=0}^{N_K} \left[(r_2 - r_1)^2 \cdot \sum_{v=0}^{N_P} \left\{\mathbf{pdf}\left(r_1; N_K, \mathbf{ccdf}(t; v, c)\right) \cdot \mathbf{pdf}(r_2; N_K, \mathbf{ccdf}(t; v, c)) \cdot \mathbf{pdf}(v; N_P, p)\right\}\right]$$

Expected distance between total KC responses in two different individuals for two different odors ($\boldsymbol{E[D_2]}$)

For two different individuals (A and B) responding to two different odors (1 and 2), D_2 is either $(\kappa^{(A,1)} - \kappa^{(B,2)})^2$ or $(\kappa^{(A,2)} - \kappa^{(B,1)})^2$. Both these terms are symmetric and have the same expected value, so we show calculation only for the first term, $(\kappa^{(A,1)} - \kappa^{(B,2)})^2$. The expected value of the term can be calculated as

$$E[D_2] = \sum_{r_1=0}^{N_K} \sum_{r_2=0}^{N_K} (r_2 - r_1)^2 \cdot \Pr\left[\left(\kappa^{(A,1)} = r_1\right) \cap \left(\kappa^{(B,2)} = r_2\right)\right]$$

Here, $\kappa^{(A,1)}$ and $\kappa^{(B,2)}$ are independent of each other as both terms depend on different and independent $\vec{P}$ vectors and $\overleftrightarrow{M}$ matrices. Hence, we can rewrite the above equation as

$$E[D_2] = \sum_{r_1=0}^{N_K} \sum_{r_2=0}^{N_K} (r_2 - r_1)^2 \cdot \Pr\left[\kappa^{(A,1)} = r_1\right] \cdot \Pr\left[\kappa^{(B,2)} = r_2\right]$$

Using expressions for total KC response obtained earlier,

$$E[D_2] = \sum_{r_1=0}^{N_K} \sum_{r_2=0}^{N_K} \left[(r_2 - r_1)^2 \cdot \left(\sum_{v=0}^{N_P} \mathbf{pdf}\,(r_1; N_K, \mathbf{ccdf}(t; v, c)) \cdot \mathbf{pdf}(v; N_P, p) \right) \cdot \left(\sum_{v=0}^{N_P} \mathbf{pdf}\,(r_2; N_K, \mathbf{ccdf}(t; v, c)) \cdot \mathbf{pdf}(v; N_P, p) \right) \right]$$

Calculations using the above formulae

These formulae enable direct calculation of $E[D_1]$, $E[D_2]$, and $E[S]$ without simulations. In **Supplementary Fig. 5a**, we show the values of $E[D_1]$ and $E[D_2]$ as a function of the total number of KCs in the model for *Drosophila* mushroom body ($N_P = 50$, $p = 0.5$, $c = 0.14$, and $q = 0.1$). When number of KCs ($\mathrm{N_K}$) is 2000, corresponding to the *Drosophila* mushroom body, $\mathrm{E[D_1]}$ and $\mathrm{E[D_2]}$ are 2.14×10^2 and 1.14×10^4, respectively, resulting in a stereotypy value (E[S]) of 0.96.

Expected distances when PN responses to one odor are obtained by shuffling responses to another odor

In our binary framework, PN responses to two odors are shuffled versions of each other if they have the same number of active PNs. Specifically, for two odors 1 and 2,

$$\mathbf{ones}(\vec{P}) \equiv \mathbf{ones}(\overrightarrow{P^{(1)}}) = \mathbf{ones}(\overrightarrow{P^{(2)}})$$

This constraint does not affect the calculations for $E[D_1]$ as it is calculated between two individuals for the same odor. $E[D_2]$ can be calculated as

$$E[D_2] = \sum_{r_1=0}^{N_K} \sum_{r_2=0}^{N_K} (r_2 - r_1)^2 \cdot \Pr\left[(\kappa^{(A,1)} = r_1) \cap (\kappa^{(B,2)} = r_2) \right]$$

Note that $\kappa^{(A,1)}$ and $\kappa^{(B,2)}$ cannot be assumed independent because both the odors, 1 and 2, have the same number of active PNs. By marginalization over this number,

$$\Pr\left[(\kappa^{(A,1)} = r_1) \cap (\kappa^{(B,2)} = r_2) \right] = \sum_{v=0}^{N_P} \left(\Pr\left[(\kappa^{(A,1)} = r_1) \cap (\kappa^{(B,2)} = r_2) \mid \mathbf{ones}(\vec{P}) = v \right] \cdot \Pr\left[\mathbf{ones}(\vec{P}) = v \right] \right)$$

Now, for a given number of $1s$ in $\overrightarrow{P^{(1)}}$ and $\overrightarrow{P^{(2)}}$, the total responses of KCs in the two individuals become independent as noted earlier. Therefore,

$$\begin{aligned} \Pr\left[(\kappa^{(A,1)} = r_1) \cap (\kappa^{(B,2)} = r_2) \mid \mathbf{ones}(\vec{P}) = v \right] &= \Pr\left[\kappa^{(A,1)} = r_1 \mid \mathbf{ones}(\overrightarrow{P^{(1)}}) = v \right] \cdot \Pr\left[\kappa^{(B,2)} = r_2 \mid \mathbf{ones}(\overrightarrow{P^{(2)}}) = v \right] \\ &= \mathbf{pdf}(r_1; N_K, \mathbf{ccdf}(t; v, c)) \cdot \mathbf{pdf}(r_2; N_K, \mathbf{ccdf}(t; v, c)) \end{aligned}$$

Putting it all together,

$$E[D_2] = \sum_{r_1=0}^{N_K} \sum_{r_2=0}^{N_K} \left[(r_2 - r_1)^2 \cdot \sum_{v=0}^{N_P} \{ \mathbf{pdf}(r_1; N_K, \mathbf{ccdf}(t; v, c)) \cdot \mathbf{pdf}(r_2; N_K, \mathbf{ccdf}(t; v, c)) \cdot \mathbf{pdf}(v; N_P, p) \} \right]$$

Thus, $E[D_2] = E[D_1]$, and therefore stereotypy is equal to 0 when the PN responses to the two odors are shuffled versions of each other.

Supplementary references

1. Schaffer, E. S. *et al.* Odor Perception on the Two Sides of the Brain: Consistency Despite Randomness. *Neuron* **98**, 736-742.e3 (2018).

2. Jortner, R. A. Network architecture underlying maximal separation of neuronal representations. *Front. Neuroeng.* **5**, 19 (2013).
